# Lighting up Individual Organelles With Fluorescent Carbon Dots

**DOI:** 10.3389/fchem.2021.784851

**Published:** 2021-11-26

**Authors:** Haifang Liu, Jiancheng Guo, Aaron Albert Aryee, Linlin Hua, Yuanqiang Sun, Zhaohui Li, Jianbo Liu, Wenxue Tang

**Affiliations:** ^1^ Precision Medicine Center of the Second Affiliated Hospital of Zhengzhou University, Zhengzhou, China; ^2^ College of Chemistry of Zhengzhou University, Zhengzhou, China

**Keywords:** carbon dots, organelle targeted, cell imaging, fluorescence, precursors

## Abstract

Cell organelles play crucial roles in the normal functioning of an organism, therefore the disruption of their operation is associated with diseases and in some cases death. Thus, the detection and monitoring of the activities within these organelles are of great importance. Several probes based on graphene oxide, small molecules, and other nanomaterials have been developed for targeting specific organelles. Among these materials, organelle-targeted fluorescent probes based on carbon dots have attracted substantial attention in recent years owing to their superior characteristics, which include facile synthesis, good photostability, low cytotoxicity, and high selectivity. The ability of these probes to target specific organelles enables researchers to obtain valuable information for understanding the processes involved in their functions and/or malfunctions and may also aid in effective targeted drug delivery. This review highlights recently reported organelle-specific fluorescent probes based on carbon dots. The precursors of these carbon dots are also discussed because studies have shown that many of the intrinsic properties of these probes originate from the precursor used. An overview of the functions of the discussed organelles, the types of probes used, and their advantages and limitations are also provided. Organelles such as the mitochondria, nucleus, lysosomes, and endoplasmic reticulum have been the central focus of research to date, whereas the Golgi body, centrosome, vesicles, and others have received comparatively little attention. It is therefore the hope of the authors that further studies will be conducted in an effort to design probes with the ability to localize within these less studied organelles so as to fully elucidate the mechanisms underlying their function.

## Introduction

The eukaryotic cell contains numerous organelles, such as the nucleus, lysosomes, Golgi body, and mitochondria, which all play critical roles in the normal functioning of biological systems. For instance, mitochondria are often regarded as the powerhouse of the cell and support a diverse range of cellular processes mediated by adenosine triphosphate (ATP), including biomolecule synthesis, cell division, and so on (Newmeyer and Ferguson Miller, 2003; Lodish et al., 2008). The nucleus, which can be considered the heart of the cell, stores the hereditary material (DNA) as well as coordinating certain cellular processes such as growth, protein synthesis, and reproduction (Dean and Hinshelwood, 1963). The biomolecules present in cell organelles have also been observed to play key roles in their overall function. Furthermore, the difference in the signal of these biomolecules has been found to be influenced by their location in the cell; thus, the ability to track their activities within specific organelles is very important as it provides the opportunity to understand the basic function of the organelle as well as lead to improvements in the treatment of the associated diseases (Alamudi et al., 2016).

In recent years, several techniques have been developed for monitoring various activities within the cell, among which fluorescent labelling has received the most attention. Fluorescent labelling is a powerful tool for examining the localization and movement of biomolecules in cellular processes owing to its ability to afford non-invasive real-time signals with high spatial resolution and sensitivity, alongside additional advantages such as facile preparation, low cost, and high selectivity (D'Angelis do Barbosa et al., 2015; Alamudi et al., 2016; Zhu et al., 2016; Gao et al., 2019; Liu and; Gao et al., 2020; Chen et al., 2020; Liu et al., 2019a; Yang et al., 2020; Zhu et al., 2019; Zlitni et al., 2020). In this regard, numerous fluorescent probes, such as small molecules (D'Angelis do Barbosa et al., 2015; Liu et al., 2020), quantum dots (Derfus et al., 2004; Lovrić et al., 2005), nanodots (Mao et al., 2016; Hua et al., 2018a), and carbon dots (CDs) (D'Angelis do Barbosa et al., 2015; Chen et al., 2019; Liu et al., 2019a), have been designed and synthesized for the monitoring of subcellular activities.

As a new kind of 0D carbon nanomaterial, CDs have emerged as a novel type of photo luminescence nanomaterial, which has attracted significant and growing interest in both scientific and technical area. CDs are made from carbon, which are the basic building block of life itself, that is their important distinction. Based on this simple fact, CDs have demonstrated better biocompatibility in many tested cell lines, mice and zebrafish compared with most inorganic nanoparticles and organic small molecule probes. The most striking property of CDs is the excitation-dependent photoluminescence, which arises from the quantum confinement effects, the large rigid *p*-conjugated structure and the presence of surface defects. (Yuan et al., 2016). Currently, the fluorescence emission wavelength of CDs can be adjusted from blue to far red by different synthesis methods (Zheng et al., 2016) and via the use of modification agents, (Ding et al., 2016). This distinct property makes CDs favourable in bioimaging applications (Wang et al., 2011). Compared with commercially available organic dyes, CDs-based probes have been reported to exhibit some unique properties, such as high photostability, facile design and synthesis methods, low cytotoxicity, and good aqueous solubility. Based on these excellent features, the application of CDs have thus emerged as a powerful tool for organelle monitoring (Liu et al., 2019a; Chen et al., 2019).

**FIGURE 1:**
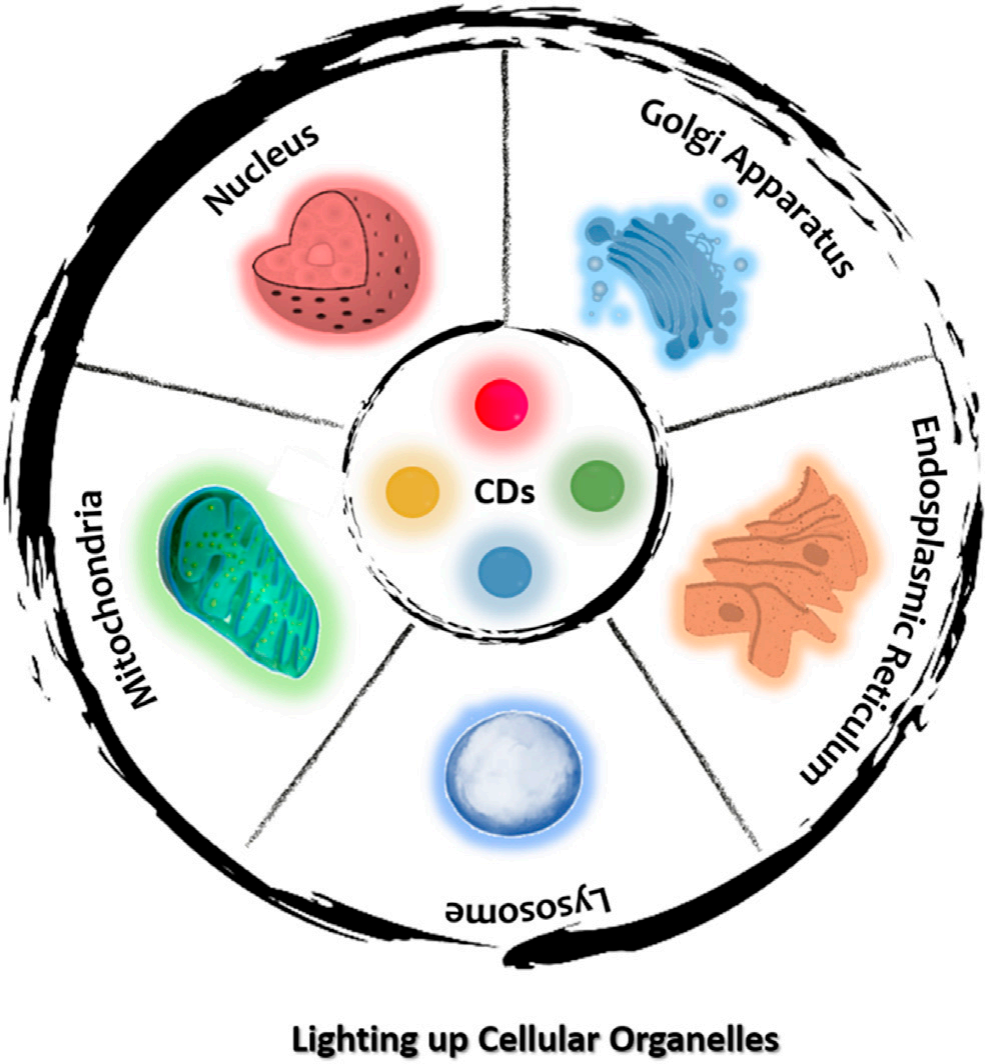
Schematic diagram for specifically lighting up individual organelles using CDs.

This review aims to provide an up-to-date summary of CDs-based organelle-specific probes, which are grouped by their target organelle so as to highlight the underlying design strategies and recent progress made in this field (Figure 1). The precursors of CDs are first highlighted in this review because the ability of some CDs to exhibit intrinsic organelle targeting has been observed to originate from its precursors. Herein, we present some information on the latest probes based on CDs and their application in imaging specific organelle, such as nucleus, mitochondria, lysosomes, and endoplasmic reticulum, Golgi body. For each organelle, a brief overview of its composition and functions is first provided, followed by a discussion of the properties of the corresponding probes, their advantages and limitations, and opportunities for future research. Some applications of these CDs-based organelle-specific probes are also presented. Next, we discuss the CDs which can migrate between subcellular organelles. These CDs could enrich our understanding of how CDs interact with cells, and shed new lights on the design of novel CDs for biomedical applications. Finally, we discuss the challenges in this field and share some possible future directions.

## Nucleus

The nucleus, which is enclosed within a double membrane referred to as the nuclear envelope, can be regarded as the control center of the cell owing to its function of regulating gene expression (Liu et al., 2019a). The nucleus is known to contain DNA, RNA and various proteins. The phospholipid bilayer nature of the nuclear membrane prevents the movement of large molecules into the nucleus but allows for the transmembrane diffusion of small nonpolar molecules (Dean and Hinshelwood, 1963; Lodish et al., 2019). Abnormalities in the nuclear morphology and envelope have been linked to many diseases and conditions, including ageing (Stephens et al., 2018), heart disease (Butin Israeli et al., 2012), skeletal myopathies (Burke and Stewart, 2002), cancer (Reddy and Feinberg, 2013), progeria (Papanicolaou and Traut, 1997), Alzheimer’s disease (Su et al., 1994), and so on. Therefore, targeted imaging of the nucleus is essential for the early diagnosis of these conditions. CDs can be classified as nanomaterials with dimensions of 1–10 nm. This relatively small size of CDs serves as an additional advantage for their application as nucleus-specific probes as they can penetrate the nuclear membrane (Liu et al., 2019a; Wu et al., 2021). Also, the surface charge on CDs has been observed to be a crucial factor in nucleus-targeted imaging (Wang et al., 2018).

### Modified CDs for Nucleus-Targeted Imaging

In the past, nucleus-targeted imaging was predominantly accomplished by the attachment of nuclear localization signal (NLS) peptides to the positively charged surface of CDs (Zhu et al., 2019). NLS peptides are composed of functional amino acid sequences whose functions are dependent on their sequence and structure (Roy et al., 2017). The ability of NLS peptides to target the nucleus has been demonstrated to originate from their interactions with importin within the nuclear pore complex. Several studies have been conducted based on the use of NLS peptides for nucleus-targeted imaging and related applications. For instance, Yang and Wang et al. synthesized blue-emitting CDs (average diameter = 5.6 nm, λ_abs_ = 350 nm, λ_em_ = 450 nm) using citric acid, PEG5000, and ethylenediamine as precursors via hydrothermal treatment (Yang et al., 2016). The CDs were then coupled with an NLS peptide (PKKKRKVG) via EDC/NHS conjugation to realize the nucleus-targeted imaging of MCF7 and A549 cells. The authors observed that in the absence of the NLS peptide the CDs were mainly localized in the cytoplasm. In contrast, the conjugation of the NLS peptide enabled nucleus-targeted imaging, which was also influenced by the small size of the CDs (<10 nm).

Another study by Gao and Yang et al. utilized an NLS peptide to achieve nuclear targeting by carbon quantum dots (CQDs; average diameter = 3.75 nm, λ_abs_ = 479 nm, λ_em_ = 546 nm) functionalized with azido molecular beacon DNA by a copper(I)-catalysed alkyne–azide cycloaddition reaction (Gao M. X. et al., 2017). The CQDs were prepared from 3,4,9,10-perylenetetracarboxylic dianhydride, which endowed the as-synthesized CQDs with carboxyl functional groups. The authors observed that the functionalization of the CQDs with gold nanoparticles (AuNPs) and DNA resulted in a higher migration rate with respect to AuNPs functionalized with DNA. They concluded that this was attributable to the difference in charge-to-mass ratio resulting from the different conjugation strategies. Confocal microscopy images confirmed the ability of the NLS-CQDs to localize within the nucleus of cells, which was ascribed to the conjugation with the NLS peptide as without it the alkynylated CQDs did not exhibit nuclear staining.

**FIGURE 2:**
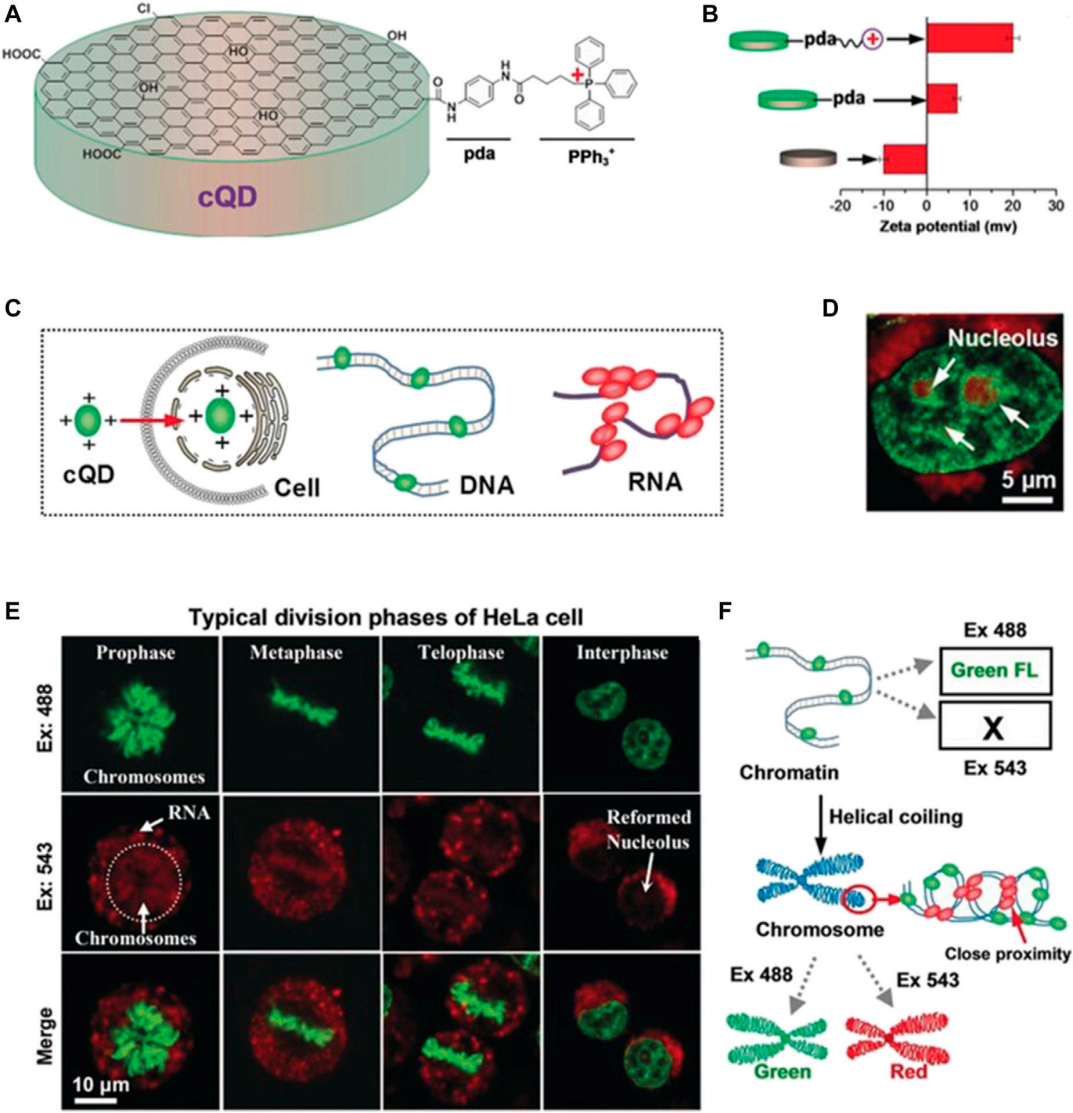
**(A)** Schematic diagram showing the synthesis of a nucleus-targeted CQD probe **(B)** Zeta potentials of the CQDs with various chemical modifications **(C)** Schematic diagram showing the interactions of the CQD probe with DNA and RNA **(D)** Fluorescence micrographs of HeLa cells stained with the CQDs upon excitation at 488 and 543 nm **(E)** Confocal images of HeLa cells at various stages of the cell cycle **(F)** Determination of the nature of DNA structures based on the emission of green or red fluorescence. Reproduced with permission from Han et al., 2019. Copyright (2019) Wiley-VCH.

In 2019, Zhang et al. developed a novel nuclear targeting probe based on 4-carboxybutyltriphenylphosphonium (PPh_3_^+^) bromide and CQDs (Figure 2) (Han et al., 2019). In addition to the excellent properties displayed by this probe, it could also distinguish between RNA and DNA, thus providing valuable and specific information concerning the nuclear activities. The zeta potential, diameter, and absorption and emission wavelengths of this nanoprobe were reported to be +20 mV, ∼3 nm, 265 nm, and 510 nm, respectively. Confocal images revealed that the interactions of this nanoprobe with dsDNA and ssRNA resulted in the emission of green fluorescence and red fluorescence, respectively, upon incubation with HeLa cells, thus providing a basis for their differentiation.

### CDs With Intrinsic Nuclear Targeting Properties

In recent years, CDs with intrinsic nuclear targeting properties have also been synthesized, thus expanding the horizon for CDs-based probes for such purposes. These probes are typically based on the use of substances possessing hydrophobic properties. For example, Zhu et al. developed nucleoli-specific CDs-based probes with tunable surface charge properties that could be varied by altering the molar ratio of the precursors, i.e. citric acid (CA) and ethanediamine (EDA) (Zhu et al., 2019). The authors observed that the surface charge increased (from −17.9 to −2.84 mV) as the molar ratio of CA:EDA was decreased (from 1:0.5 to 1:3) with a concomitant enhancement in the staining of nucleoli. This enhancement was ascribed to the higher cellular uptake of the CDs with higher zeta potential.

Recently, novel negatively charged CDs with intrinsic nuclear targeting properties were synthesized by Hua et al. and applied to the delivery of protoporphyrin into the nucleus (Hua et al., 2018b)]. The CDs were prepared from *m*-phenylenediamine and l-cysteine through one-pot hydrothermal treatment. Characterization of the CDs revealed an average diameter of 3.8 ± 0.5 nm, two absorption peaks at 269 and 405 nm, and an emission peak at 520 nm. Confocal images showed that the probe was able to stain the nuclei of two cancer cell lines (HepG2 and A549) and three normal cell lines (RAW264.7, AT II, and L02) to afford green fluorescence. However, owing to the structure of nucleic acids, which are rich in negatively charged phosphate units, it is generally difficult for neutral and negatively charged molecules to pass through the nuclear membrane (Xu et al., 2007; Cai et al., 2017). In contrast, positively charged CDs have been observed to circumvent this challenge as a result of their ability to evade the endo/lysosomes through the so-called sponge effect (Mohammadinejad et al., 2019). Therefore, CDs possessing a positive charge (zeta potential) have been applied extensively for nuclear targeting. The design strategy for forming these cationic CDs usually involves passivation with substances bearing amino groups (Li et al., 2018). However, factors such as their facile removal from the cytoplasm and unfavourable interactions with negatively charged intracellular proteins may result in severe serum inhibition, which greatly limits *in vivo* applications (Vankayala et al., 2015).

**FIGURE 3:**
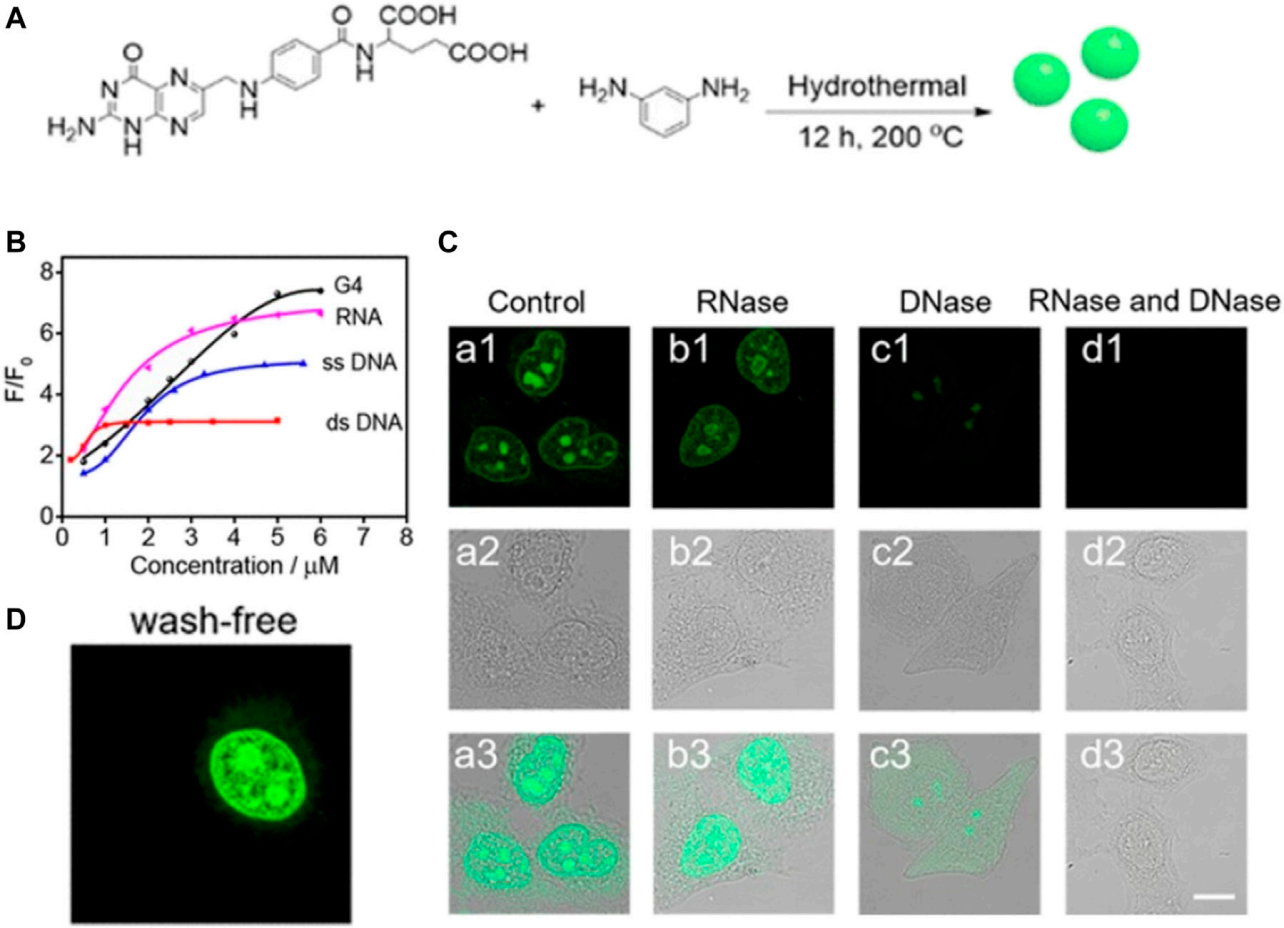
**(A)** Schematic diagram showing the synthesis of HBIE-CDs **(B)** Fluorescence spectra of the HBIE-CDs in water, dsDNA solution, RNA solution, DNase solution, and RNase solution **(C)** Washing-free fluorescence images of HeLa cells stained with HBIE-CDs **(D)** Confocal image of HeLa cells stained with HBIE-CDs and further incubated with RNase and DNase, scale bar = 20 μm. Reproduced with permission from Liu and Yang et al., 2019. Copyright (2019) American Chemical Society.

For the purpose of bioimaging, the fluorescence quantum yield of CDs is an important factor; hence, fluorophores with longer wavelengths, brighter fluorescence, and high quantum yield are generally preferred (He et al., 2016). Hydrogen bonding has been observed to play significant roles in biological fluorophores as it can inhibit nonradiative processes to afford a high fluorescence quantum yield. On the basis of this principle, Liu and Yang et al. synthesized novel DNA- and RNA-sensitive hydrogen-bond-induced emission CDs (HBIE-CDs) (Liu et al., 2019a). The CDs were fabricated by a hydrothermal method using *m*-phenylenediamine and folic acid as precursors, which have the ability to bind nucleobases at multiple sites. This HBIE-based sensing mechanism can potentially be generalized for the fabrication of sensors displaying fluorescence turn-on detection. The average size, absorption wavelength, and emission wavelength of the HBIE-CDs were observed to be 2.6, 394, and 535 nm, respectively. The as-synthesized HBIE-CDs displayed several excellent characteristics, such as longer-wavelength emission upon hydrogen bond formation and the ability to mediate washing-free subcellular labelling and imaging of the nucleus in living cells (Figure 3). These CDs have huge potential in clinical diagnosis for imaging of the nuclear morphology.

Red-emitting CDs capable of nuclear targeting were synthesized by Hua et al. using *p*-phenylenediamine and doped with Ni by a hydrothermal method (Hua et al., 2019). This probe was observed to exhibit a quantum yield of 45.6% (in DMSO) as well as good photostability. The size, absorption wavelength, and emission wavelength of these CDs were reported to be approximately 2.9, 510, and 605 nm, respectively. The authors proposed that Ni^2+^ ions could be beneficial for promoting the formation of CDs and realizing higher surface oxidation. The CDs were also observed to be sensitive towards RNA and DNA, enabling them to be used to monitor these components within the nucleolus.

Malfunctioning of the cell nucleus has also been associated with disorders such as Down syndrome, cystic fibrosis, and leukaemia (Xiang et al., 2012; McKiernan et al., 2013). Thus, there is substantial demand for probes that are able to specifically locate the nucleus and can be applied as biosensors and theranostic agents for drug delivery. In recent years, various probes capable of serving as carriers for drug delivery into the nucleus have been developed, with doxorubicin (Dox) frequently employed as the model drug (Wang et al., 2013; Jung et al., 2015; Jia et al., 2016; Yang et al., 2016; Yao et al., 2016; Wang et al., 2017; Yao et al., 2017; Hua et al., 2018a; Pei et al., 2018; Li et al., 2019). The conjugation of Dox to CDs is usually accomplished through π–π* stacking and electrostatic interactions (Yang et al., 2016).

As an alternative method, Yang and Wang et al. achieved the nuclear staining of A549 cells within 4 h using NLS-modified CDs covalently conjugated with Dox, as mentioned in Section 2.1 (Yang et al., 2016). Similar results have been reported by Yao et al. and Yuan et al. The former group synthesized Gd (III)-doped CDs using citric acid and GdCl3 prior to encapsulating a high concentration of Dox (Yao et al., 2016). Characterization of the CDs revealed an average diameter of 5 nm, an absorption peak at 360 nm, and an emission peak at 485 nm. The CDs were applied as both an MRI contrast agent as well as a carrier agent for the delivery of Dox into the nucleus of MCF-7 cells. This conjugated system was able to localize in the nucleus within 2 h. Meanwhile, Yuan et al. synthesized CDs from milk and subsequently conjugated them with Dox (Li et al., 2019). Characterization of the CDs revealed an average diameter of 20 nm, an absorption peak at 290 nm, and an emission peak at 444 nm. This system was reported to exhibit enhanced antitumour behaviour towards ACC-2 cells, with confocal images confirming localization in the nucleus within 4 h.

In summary, previous nucleolus-targeted results based on CDs could be mainly attributed to the accumulation of the CDs in the cell nucleus through various noncovalent interactions between the nanoparticles and nucleic acid, such as π−π stacking, electrostatic interaction, and hydrogen bonding. Nucleus staining CDs have great potential to reveal the biological studies in cellular metabolism, growth, differentiation and heredity, and also clinical diagnosis which are associated with the changes of nuclear morphology or microenvironment.

## Mitochondria

Mitochondria are often regarded as the powerhouse of cells. The primary function of these rod-shaped organelles is converting oxygen and nutrients into ATP. In addition, mitochondria are also involved in a diverse variety of cellular processes, such as metabolism, cell division, signal transduction, and so on (Ahn et al., 2018; Yang et al., 2018). The dysfunction of these processes is associated with neurodegenerative diseases, such as Parkinson’s disease and Alzheimer’s disease (Chan, 2006; Lin and Beal, 2006). The generation of ATP in the mitochondria during cellular respiration results in the mitochondrial membrane possessing a negative potential (Modica-Napolitano and Aprille., 2001; Modica-Napolitano and Singh, 2004; Walker and Dickson., 2006; Lin et al., 2018). Hence, one strategy for targeting the mitochondria is the use of fluorophores bearing positively charged lipophilic moieties, such as methylpyridinium cations, triphenylphosphonium (TPP) cations, and indolium cations, which are attracted by the negative membrane potential (Ross et al., 2004).

### TPP Based Mitochondria Targeted Imaging

**FIGURE 4:**
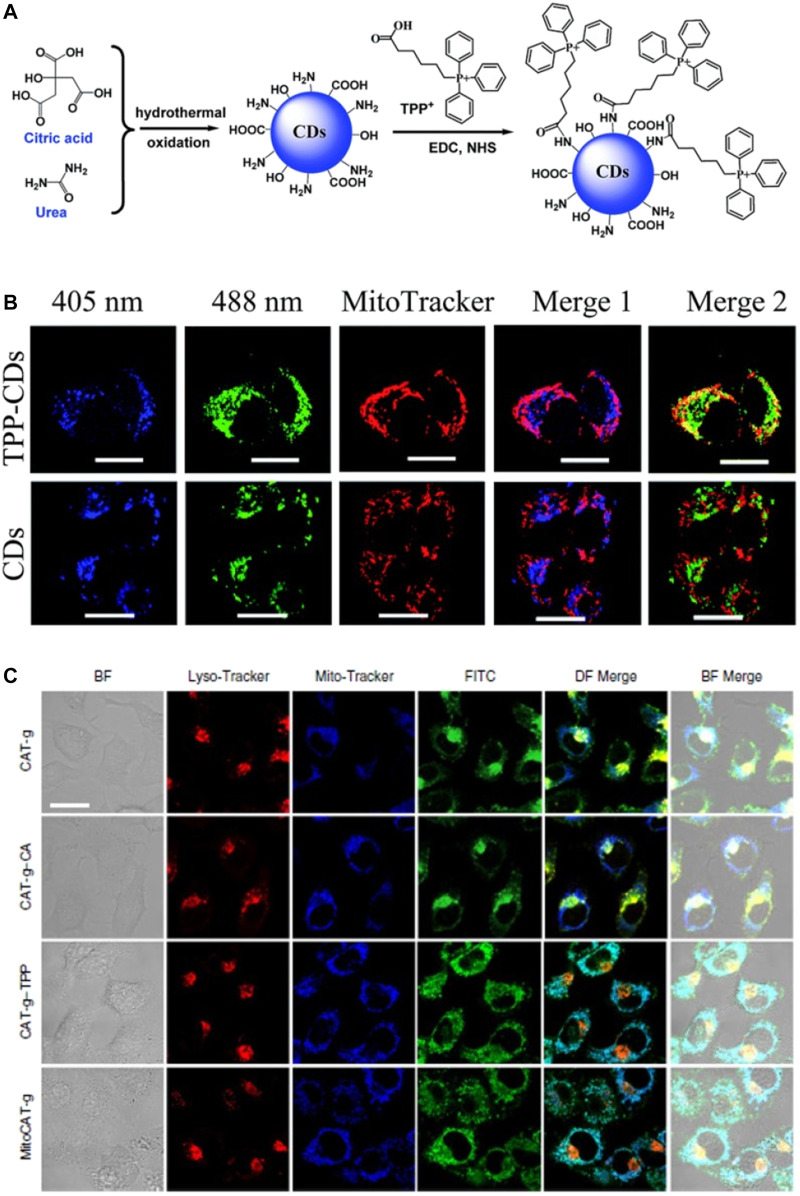
**(A)** Design concept for the TPP-CDs **(B)** Subcellular localization of TPP-CDs and CDs after 4 h incubation with HeLa cells, scale bar = 20 μm. Reproduced with permission from Wang et al., 2014. Copyright (2014) The Royal Society of Chemistry **(C)** Confocal images showing HepG2 cells after treatment with various probes, and bio-TEM images of HepG2 cells after treatment with MitoCAT-g, scale bar = 20 μm. Reproduced with permission from Gong et al., 2019. Copyright (2019) Nature Publishing Group.

Ligands bearing TPP cations have been widely used for targeting mitochondria owing to the delocalized nature and high lipophilicity of these cations (Wang et al., 2014; Zhang et al., 2015; Dong et al., 2016; Hua et al., 2017; Wu X. et al., 2017; Zhang et al., 2017; Gong et al., 2019; Zhou et al., 2019). In 2014, Wang et al. synthesized fluorescent TPP-conjugated CDs (TPP-CDs) by a hydrothermal method that could target mitochondria (Figure 4A) (Wang et al., 2014). Characterization of the TPP-CDs revealed an average diameter of 8.5 nm, an absorption peak at 340 nm, and an emission peak at 425 nm. The TPP-CDs exhibited low cytotoxicity and were biocompatible at incubation times of 8 and 6 h, respectively. The CDs were synthesized from urea and citric acid using a one-pot hydrothermal method. These precursors endowed the obtained CDs with various functional moieties including–NH_2_, –COOH, and–OH groups, enabling the conjugation of TPP via amidation between TPP-COOH and the NH_2_-bearing CDs. After conjugation with TPP, the zeta potential of the CDs increased remarkably. Two-photon confocal microscopy analysis demonstrated the selective accumulation of the TPP-CDs in the mitochondria and a high two-photon fluorescence signal around the nucleus.

In a recent study, Zhou et al. developed a ratiometric nanosensor suitable for tracking ^•^OH radicals in the mitochondria (Zhou et al., 2019). This nanosensor was based on CDs synthesized via the microwave-assisted hydrothermal treatment of 1,2,4-triaminobenzene and formamide followed by conjugation with coumarin-3-carboxylic acid (CCA) and (4-carboxybutyl) triphenylphosphonium bromide. In this system, the CCA moiety served as the recognition site for ^•^OH radicals, switching from non-fluorescent to strong blue fluorescence upon reaction with ^•^OH. The zeta potential of the nanosensor was observed to be more positive (+19.6 mV) compared to that of the CDs (+3.2 mV), thus making it more suitable for mitochondrial targeting. The blue fluorescence emission of the sensor increased upon the addition of ^•^OH within the concentration range from 0.1 to 160 μM, and the detection limit of the sensor was reported to be 70 nM. Confocal images of RAW 264.7 cells stained with the nanosensor revealed successful mitochondrial localization within 4 h. Further treatment with the CDs via different systems showed changes in the fluorescence signal based on the concentration of ^•^OH.

The mitochondria are the major cellular source of reactive oxygen species (ROS) (Dickinson and Chang., 2011). These species may originate as normal byproducts of mitochondrial processes and have also been implicated in certain pathological processes (James et al., 2007). Hence, the detection of these ROS and determination of their specific sites of origin within the cells are crucial. In this regard, Wu et al. prepared CDs for the detection of ONOO^−^ in the mitochondria (Wu L. et al., 2017). The CDs were synthesized from *o*-phenylenediamine and TPP and exhibited two absorption bands characteristic of CDs prepared from amine precursors. In the presence of ONOO^−^, the fluorescence of the CDs was quenched by photoinduced electron transfer, and benzotriazole structures were formed on the surface of the CDs by the reaction between the surface *o*-diaminobenzene moieties and ^•^NO_2_ produced by the decomposition of ONOO^−^. Cell imaging experiments revealed that the fluorescence of the CDs incubated with MCF-7 cells decreased after treatment with lipopolysaccharide and interferon-γ, indicating the suitability of the probe for intracellular peroxynitrite sensing. By virtue of their sensing mechanism, ratiometric probes have been found to possess some superior qualities, such as improved sensitivity and quantification.

Remarkable applications of mitochondria-targeted CDs in theranostics have emerged recently (Ge et al., 2014; Wang et al., 2014; Matai et al., 2015; Yang et al., 2015; Xiang et al., 2016a; Xiang et al., 2016b; Zhang et al., 2017; Geng et al., 2018; Hua et al., 2018b; Yang et al., 2018). Gong et al. reported the use of a mitochondria-targeted probe (MitoCAT-g) as a mitochondrial oxidative stress amplifier and a promising anticancer agent (Figure 4B) (Gong et al., 2019). The probe was prepared via a three-step approach, in which CDs were first synthesized from citric acid and polyene polyamine then loaded with atomically dispersed gold and conjugated with both cinnamaldehyde for ROS generation and TPP for mitochondrial targeting. The design of the probe was based on the depletion of mitochondrial glutathione, which leads to an increase in ROS and ultimately apoptosis. MitoCAT-g was also labelled with fluorescein isothiocyanate (FITC) for confocal fluorescence imaging of the cancer cells. The resulting images showed that MitoCAT-g had high cytotoxicity towards cancer cells but limited cytotoxicity towards normal cells, thereby confirming the design.

### Intrinsic Mitochondria-Targeting CDs

In spite of their advantages, TPP and other cationic moieties are toxic at higher concentrations and have also been implicated in haemolysis and damage to the mononuclear phagocyte system; thus, there exists the need to explore other design strategy for mitochondria targeted CDs (Guzman Villanueva et al., 2015). One such option is the use of CDs with an intrinsic ability to target mitochondria.

Hua et al. reported this phenomenon for the first time using CDs which were prepared from mercaptosuccinic acid, ethylenediamine, and chitosan via a one-pot hydrothermal treatment (Hua et al., 2017). Characterization of the CDs revealed an average size of 2.1 ± 0.3 nm, two absorption peaks at 260 and 343 nm, excitation-dependent emission in the range of 420–600 nm, and a high quantum yield of 11.8%. The CDs were also observed to possess high photostability as well as negligible cytotoxicity, thus suggesting high potential in the prolonged imaging of mitochondria.

With this inspiration, Gao and Jiang et al. synthesized CDs that exhibited intrinsic mitochondrial targeting properties as well as the ability to distinguish cancer cells from normal cells (Gao G. et al., 2017). These CDs were synthesized using glycerol and (3-aminopropyl) trimethoxysilane (APTMS) as precursors. Characterization of the CDs revealed an average size of 3.5 ± 0.5 nm, two absorption peaks at 275 and 330 nm, an emission maximum at 448 nm, and a quantum yield of 21.3%. The green CDs were found to be capable of targeting the mitochondria of various cell types with low cytotoxicity. On the basis of differences in the uptake efficiencies and mitochondrial membrane potentials, the CDs could be used to differentiate cancerous cells from normal cells.

**FIGURE 5:**
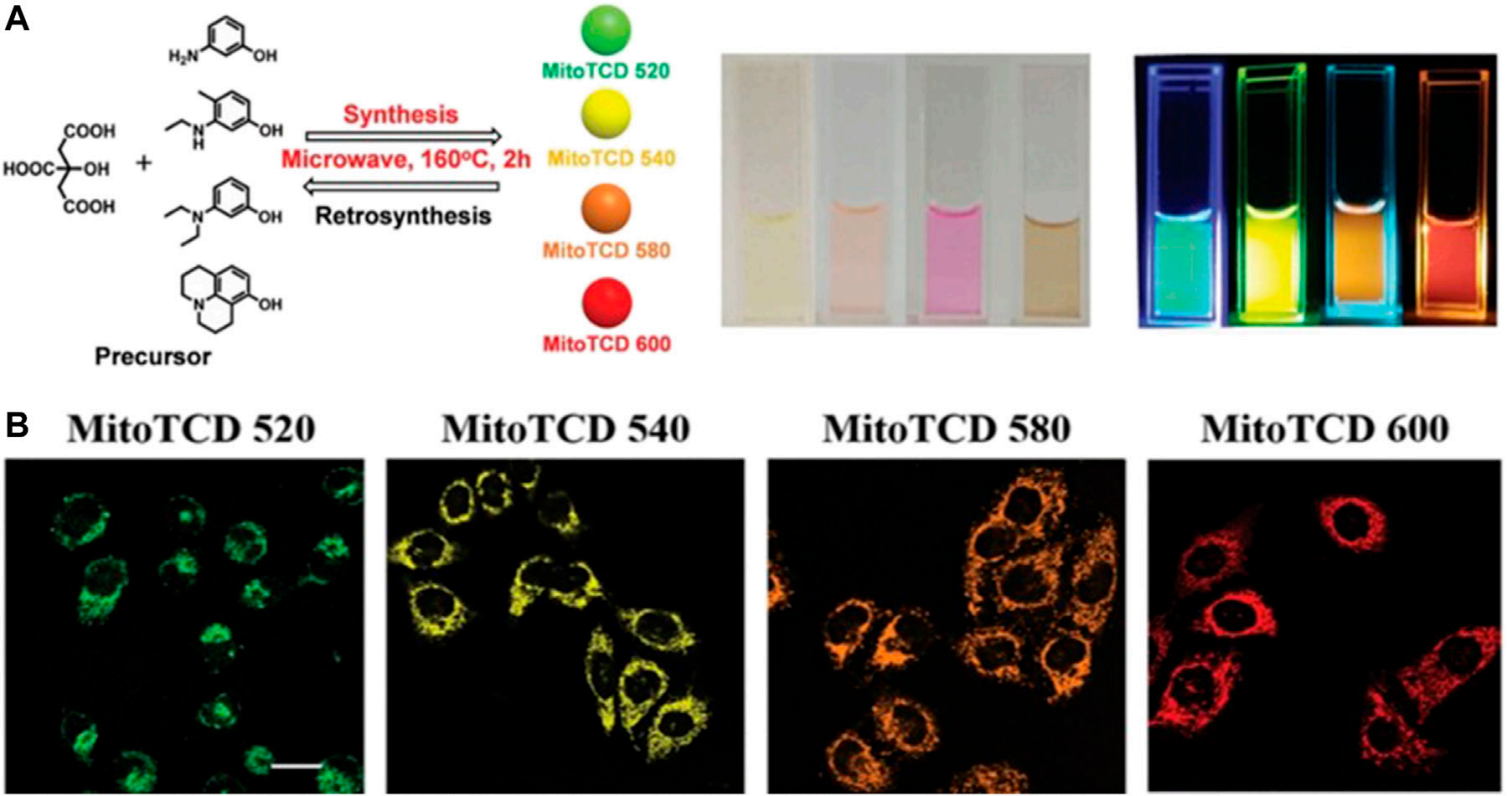
**(A)** Synthesis design and images of the mitochondria-targeted CDs **(B)** Confocal images of HeLa cells and zebrafish stained with the synthesized CDs, scale bar = 10 μm. Reproduced with permission from Geng et al., 2019. Copyright (2019) Wiley-VCH.

CDs with longer emission wavelengths are preferred for imaging applications owing to their lower incidence of photodamage to biological samples, deeper tissue penetration, and minimum interference from background autofluorescence (Guo et al., 2017). CDs displaying both intrinsic mitochondria-targeting ability as well as tuneable long-wavelength fluorescence were synthesized for the first time by Geng et al. (Geng et al., 2019). These CDs were designed by the retrosynthesis method and then prepared from *m*-aminophenol and citric acid. This design principle was introduced to CDs synthesis for the first time to overcome the difficulties associated with the precise synthesis of functional CDs. The authors also introduced the rhodamine structure as the luminescent centre and observed that the synthesized CDs displayed a bathochromic shift upon the replacement of the substituent groups (*m*-aminophenol) in the precursor (Figure 5). The positively charged rhodamine moiety allowed the CDs to target the negative transmembrane potential of the mitochondria. *In vivo* studies in zebrafish confirmed the low cytotoxicity and good biocompatibility of the probe, thus indicating potential applications in bioimaging and biodiagnosis.

In addition, the mitochondria dysfunction is associated with many diseases, such as neurodegenerative diseases, inflammation, cardiac dysfunction and diabetes. Consequently, studying on the behavior and status of mitochondria, as well as a detailed investigation of the microenvironment inside the mitochondria is crucial in monitoring the cellular fate, which has the propensity to enrich our understanding of the genesis and development process of mitochondrial diseases.

## Lysosomes

Lysosomes are specialized spherical vesicles inside cells that degrade large molecules through the action of hydrolytic enzymes, thus playing various roles in autophagy, homeostasis, plasma membrane repair, and the removal of waste substances from the cell (Weissmann, 1967; Yu et al., 2016). These enzymes are most active at the acidic pH (4–5.0) of the lysosome interior (Mindell, 2012). Thus, one reported strategy for lysosome targeting is the use of probes containing lipophilic amine moieties, which become protonated after diffusion through the lysosomal membrane, thus trapping the probe inside the lysosome and leading to its enrichment (Xu et al., 2016). Lysosomes are known to contain approximately 50 acid hydrolases that are responsible for hydrolysing various biomacromolecules, such as lipids, nucleic acids, proteins, and polysaccharides. Lysosomal malfunction or abnormal lysosome activities have been linked to various disorders, including lysosomal storage diseases, inflammation, neurodegenerative disease, and cancer (Tong et al., 2020; Li et al., 2021; Strzyz, 2021).

CDs-based probes have been shown to enter cells via the process of endocytosis, while the lysosomes playing a key role in this process via its pH value is less than that of the cytoplasm (Esteves da Silva and Gonçalves, 2011; Zhou et al., 2014). The main principle underlying this design requires probes to have a low p*K*_a_ value (Xu et al., 2016). One way of achieving lysosome targeted is the use of bearing lipophilic amines on the CDs. In this regard, the application of amino acids as passivating agents or carbon sources in the synthesis of CDs has been shown to be effective (Liu et al., 2019b; Ronzani et al., 2019; Qin et al., 2020; Sun et al., 2020).

### Morpholine-Modified CDs for Lysosome Targeting

Studies on the use of morpholine as a functional group for lysosome-targeting probes have been reported (Wan et al., 2014; Wu X. et al., 2017; He et al., 2017; Biswas et al., 2021; Mukherjee et al., 2021). For example, Wu et al. synthesized CDs functionalized with a morpholine derivative (CDs-PEI-ML) to serve as a lysosomal probe (Wu L. et al., 2017). In this study, the CDs-PEI-ML probe was prepared from citric acid and PEI via a hydrothermal method and the morpholine was then covalently attached to the CDs using EDC and NHS. Probes with morpholine groups modified typically display p*K*_a_ values of five to six, thus enabling them to be used to target lysosomes with their internal pH of 4–5. The zeta potential of the CDs was observed to change from −21.0 to +28.0 mV upon passivation with PEI, indicating the presence of positive charges on the CDs surface. Characterization of the CDs-PEI-ML probe revealed an average size of 3.15 nm, an absorption peak at 359 nm, an emission maximum at 441 nm, and a quantum yield of 37.7%. Confocal images demonstrated that the probe exhibited high photostability against bleaching, low cytotoxicity in HeLa cells, and effective lysosomal localization.

**FIGURE 6:**
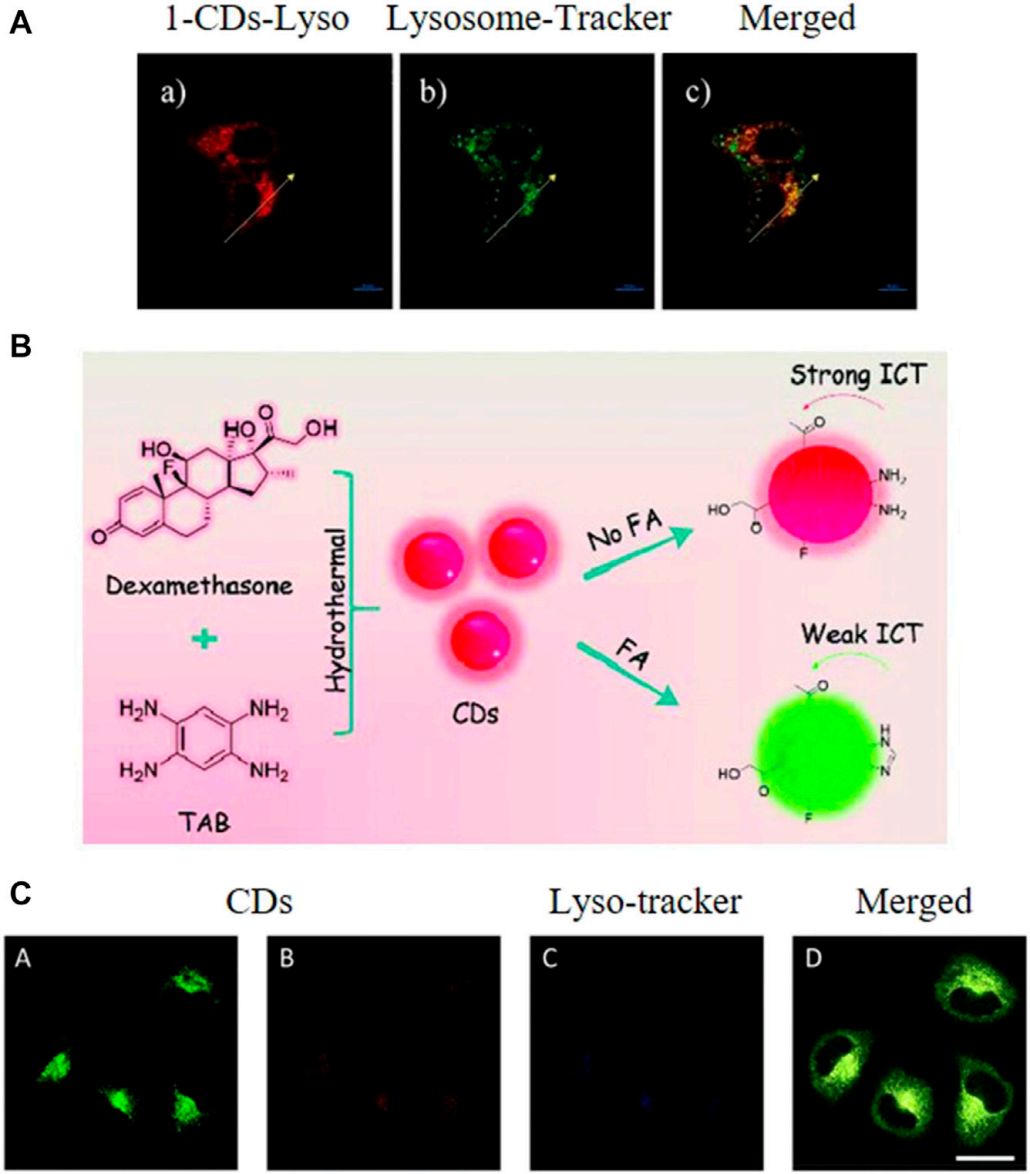
**(A)** Fluorescence images of HeLa cells stained with the synthesized CDs and a lysosome tracker, scale bar = 10 μm. Reproduced with permission from He et al., 2019. Copyright (2017) Elsevier **(B)** Synthesis of the CDs **(C)** Localization of the CDs in the lysosomes of HeLa cells, scale bar = 25 μm. Reproduced with permission from Liu et al., 2019b. Copyright (2019) The Royal Society of Chemistry.

Lysosomal pH is an important factor in its normal functioning, therefore probes capable of revealing pH fluctuations within the lysosomes offer profound benefits. In this regard, He et al. developed a ratiometric nanosensor capable of monitoring lysosomal pH (He et al., 2017). This nanosensor was based on 1,8-napthalimide derivative, morpholine, and CDs synthesized using CA and EDA as precursors by a simple hydrothermal method. In acidic medium, the nanosensor was observed to exhibit green fluorescence. However, a gradual blue shift occurred with increasing pH, resulting in the nanosensor emitting blue fluorescence (Figure 6A). The nanosensor was reported to display a linear range for pH sensing of 5.6–7.4. In addition, confocal images displayed a pH-dependent signal when the nanosensor was applied to HeLa cells.

### Lipophilic-Amine-Functionalized CDs for Lysosome Targeting

Owing to the acidotropic behaviour of weakly basic amines, probes bearing such groups can selectively accumulate inside lysosomes with their acidic interior pH (Abeywickrama et al., 2019; Fan et al., 2021). Even though probes equipped with functional groups have proved effective for lysosome targeting, their complex modification process limit their broader application. Recently, another type of CDs bearing weakly basic amino groups has been applied to lysosome targeting (Chen et al., 2018; Zhang et al., 2018; Geng et al., 2020; Gao et al., 2020; Guo et al., 2020b). These CDs combine the good biocompatibility and fluorescence properties of CDs and the lysosome targeting properties of lipophilic amines. For example, Zhang et al. synthesized highly photoluminescent emerald CDs from ethanediamine and *p*-benzoquinone and reported their suitability for lysosome targeting (Singh et al., 2019). The CDs exhibited an average size of 2.02 nm, absorption peaks at 234 and 410 nm, an emission maximum at 530 nm, a quantum yield of 15%, good aqueous solubility, pH sensitivity, and high photostability. The CDs possessed abundant amino groups, and the confocal images after incubation with A549 cells and HepG2 cells revealed a time-dependent decrease in the fluorescence emission when the probe was treated with dexamethasone to induce morphological changes within the cells.

Hydrophobic CDs capable of targeting the lysosomes were synthesized by Mao et al. using 1-ethyl-3-methylimidazolium bromide via a hydrothermal method (Mao et al., 2016). Characterization of the CDs revealed an average size of 8.52 nm, concentration-dependent absorbance and photoluminescence where the emission wavelength shifted from 450 to 540 nm, and a quantum yield of 2.5–4.8%. Furthermore, these hydrophobic CDs were found to have a rapid penetration rate as they emitted fluorescence within 1 min of entering HeLa cells. These properties make this probe very promising for bioimaging, which was further confirmed using A549, MCF-7, and HeLa cells.

CDs with intrinsic lysosome targeting and cancer targeting abilities were first synthesized by Liu and Sun et al. from 1,2,4,5-tetraaminobenzene and dexamethasone using a microwave-assisted hydrothermal method (Figure 6B) (Liu et al., 2019b). The as-synthesized CDs were reported to exhibit some excellent properties, such as longer-wavelength emission (absorption peak at 496 nm and emission peak at 585 nm), good aqueous solubility and biocompatibility, and high photostability. The *o*-diamino moieties on the CDs were demonstrated to react with formaldehyde and target lysosomes. As an abnormal formaldehyde concentration in lysosomes is closely related to Alzheimer’s disease, thus the detection of formaldehyde in lysosomes is of great significance. This CDs also exhibited a high selectivity for formaldehyde with a fast response and great changes of ratio values. The developed system was thus used for the detection of formaldehyde fluctuation in the lysosomes of living cells.

In addition, the lysosome dysfunction is associated with cell apoptosis and oncogenic transformation, which also might relate to various diseases, such as cancer and neurodegenerative diseases. Thus, tracking the behavior and status of lysosome, also a detailed investigation of the environment in lysosome is crucial to enrich our knowledge of related diseases.

## Endosplasmic Reticulum

The endoplasmic reticulum (ER) is an organelle in eukaryotic cells that is involved in the production, processing, and transport of proteins and lipids. The ER is composed of two compartments, namely, the rough ER and the smooth ER, with each playing different roles. In particular, the rough ER is involved in the secretion of proteins, whereas the smooth ER is involved in the synthesis of carbohydrates and lipids (Baiceanu et al., 2016). As described by Zhang et al., the accumulation of unfolded or misfolded proteins can disturb the normal functions of the ER, which induces ER stress or the cellular stress response (Zhang and Kaufman, 2008). One strategy for developing ER-specific probes involves targeting the ER-associated proteins.

Bao et al. synthesized a novel probe that targeted the ER (Bao et al., 2019). This probe, which the authors referred to as trace metal (M)-, N-, and O-doped carbon-dominated nanoparticles (MNOCNPs), was synthesized from *p*-phenylenediamine and selected trace metals via a one-pot hydrothermal approach followed by conjugation with PEG. The zeta potentials of these MNOCNPs were reported to be +52.7, +34.8, and +40.3 mV for NiNOCNPs, PdNOCNPs, and CuNOCNPs, respectively. Subsequent modification of the NiNOCNPs with PEG (PEG5k-NiNOCNPs) led to a change in the zeta potential from +52.7 to 31.2 mV and improved the dispersibility of the system in various solvents. This conjugated system was found to possess good photostability, low cytotoxicity, and high biocompatibility. Conjugation with FITC confirmed the co-localization of the PEG5k-NiNOCNPs in the ER region of the cell.

The pH is known to be a critical factor regulating the activity of the ER. For instance, Dong et al. observed that an acidic intracellular pH caused by the overproduction of protons could stimulate ER stress through the activation of GPR4, leading to adverse effects such as apoptosis and inflammation thus confirming their positive surface charge (Dong et al., 2017). Thus, probes that exhibit the dual functions of ER targeting and pH responsiveness are in high demand. Shuang et al. synthesized novel CDs with the ability to target ER as well as reflect fluctuations in its pH (Shuang et al., 2018). The CDs were prepared from urea and citric acid (these amine groups functionalized CDs referred to as ACDs) and then functionalized with laurylamine to realize ER targeting. The laurylamine functionalized CDs (LCDs) possessed an average size of approximately 3 nm and displayed a red shift in their n–π* transition compared to the ACDs (i.e. from 336 to 360 nm), an emission maximum of 440 nm, and a fluorescence quantum yield of 1.3%. The LCDs were sensitive to pH in the range of 6.2–7.2 with a correlation coefficient of 0.9944. This pH sensitivity of the LCDs was ascribed to the amount of laurylamine in the system. This led the authors to conclude that by regulating the surface functional groups on CDs, there exists the possibility of forming pH-sensitive probes. The results of FTIR and XPS analysis confirmed the successful functionalization. Laurylamine was also observed to endow the CDs with hydrophobic properties that enhanced their interaction with the cell membrane, resulting in endocytosis and subsequent localization in the ER, which was confirmed using endocytosis inhibitors. Furthermore, confocal images confirmed the pH sensitivity and ER-targeting properties of the probes in MCF-7 cells in the presence of chloroquine and dexamethasone.

In summary, CDs which are functionalized with amine groups are found to be effective for ER targeting; similar with lysosome-targeted CDs. However, the aggregation of the CDs inside ER and lysosome might be different because of the difference in their environment. Thus, the CDs’ fluorescence properties must be studied in detail to enhance their application for monitoring and imaging of ER.

## Golgi Body

The Golgi body (also referred to as the Golgi apparatus or Golgi complex) is the organelle responsible for processing proteins received from the ER for further transportation to other organelles such as the lysosomes, endosomes, and plasma membrane (Xu et al., 2016). During this process, most of the proteins undergo significant changes; hence, changes in the environment of the Golgi body are likely to affect its function and subsequently result in diseases (Bexiga and Simpson, 2013). Owing to the role of the Golgi body in binding proteins, it has been observed that biomolecules with abundant thiol groups can identify protein retention within the Golgi body by anchoring on the surfaces of the Golgi body, hence serving as a means for detection (Gauthier et al., 2009).

**FIGURE 7:**
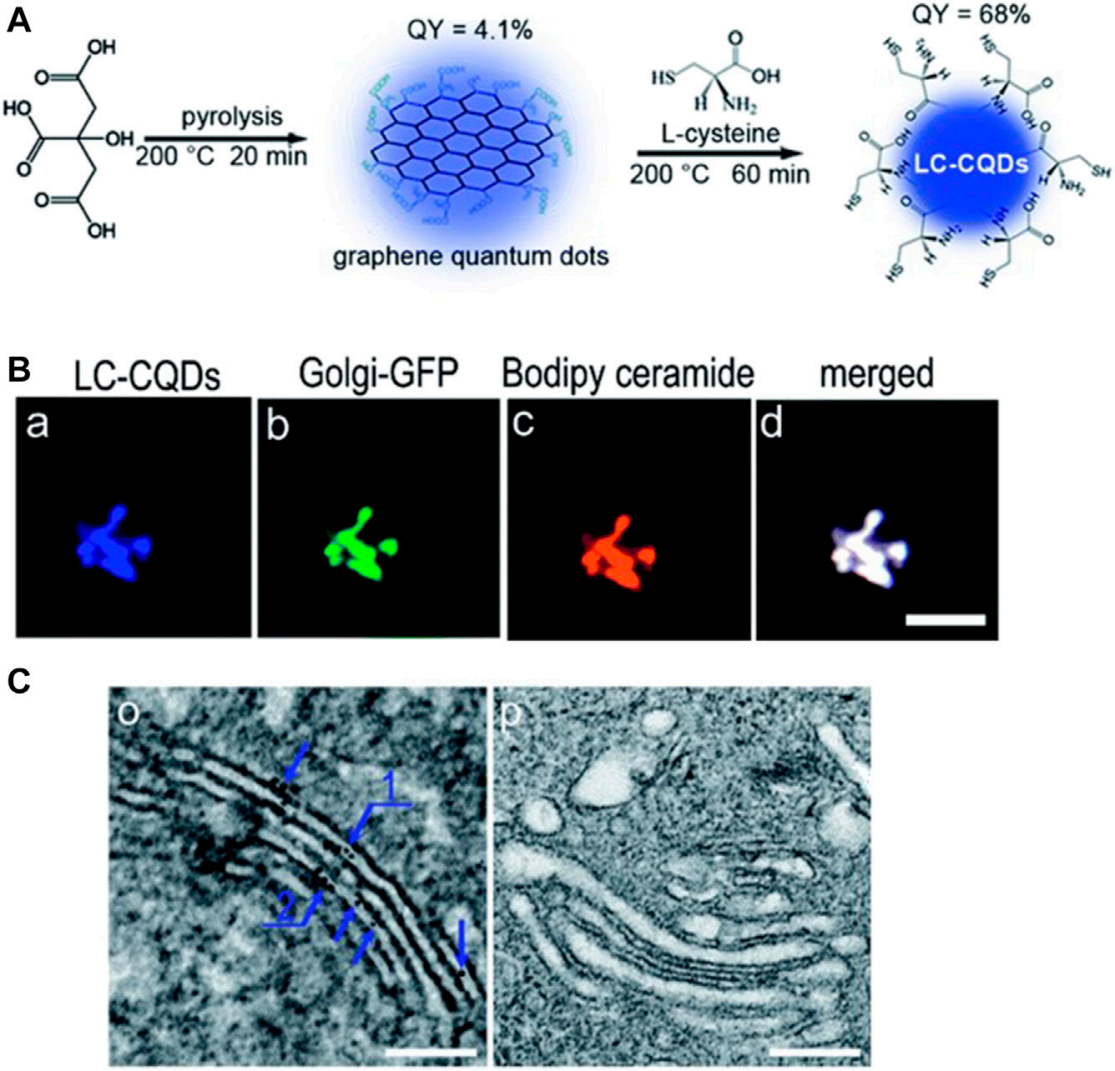
**(A)** Synthesis of CDs **(B)** Co-localization of probes within the Golgi body of HEp-2 cells, scale bar = 10 μm **(C)** TEM image of the Golgi after incubating with and without LC-CQDs, Scale bar, 200 nm. Reproduced with permission from Li et al., 2017. Copyright (2017) The Royal Society of Chemistry.

Cysteine has been found to be a useful precursor for synthesizing probes capable of staining the Golgi body due to the anchoring of galactosyltransferase and protein kinase D in the Golgi body. Inspired by this, Li et al. synthesized novel probes via pyrolysis using citric acid and l-cysteine as precursors for the imaging of the Golgi body (Li et al., 2017). The as-synthesized CDs were observed to have chiral properties, good fluorescence characteristics, and abundant cysteine residues, which has proven to be essential for Golgi targeting. Confocal images revealed that the as-synthesized probe stained the Golgi body of HEp-2 cells after 4 h of incubation and also did not affect the function of the Golgi body (Figure 7). In a further study conducted by the same authors (Li et al., 2019), these CDs were conjugated with the ricin A-chain of (CD-RTA) and found to be effective carriers for the delivery of ricin A protein into the Golgi body, thus demonstrating the potential of such CDs for selective drug delivery.

Yuan et al. synthesized optically active light-emitting carbon nanoparticles that were intrinsically capable of localizing within the Golgi body (Yuan M. et al., 2017). The CDs were synthesized from CA and d/l-penicillamine via pyrolysis. Characterization of the CDs revealed a typical size of 1–4 nm, absorption peaks at 247 and 344 nm, and the emission of blue fluorescence upon excitation at 363 nm. Further studies demonstrated that the CDs had excellent cellular uptake properties, high photoluminescence quantum yield, and low cytotoxicity as well as effectively staining the Golgi body. The authors concluded that the vicinal effect resulting from the achiral core of citric acid with the chiral penicillamine ligands in an achiral adsorption pattern induced chirality in the as-synthesized CDs via optically active surface passivation, while the abundance of thiol groups on the CDs surface enabled detection of the Golgi body. Although various CDs synthesised from precursors containing L-stereo functional groups were used for Golgi targeted imaging, a clear mechanism for aggregation in Golgi body is lacking.

## Translocation Between Organelles

**FIGURE 8:**
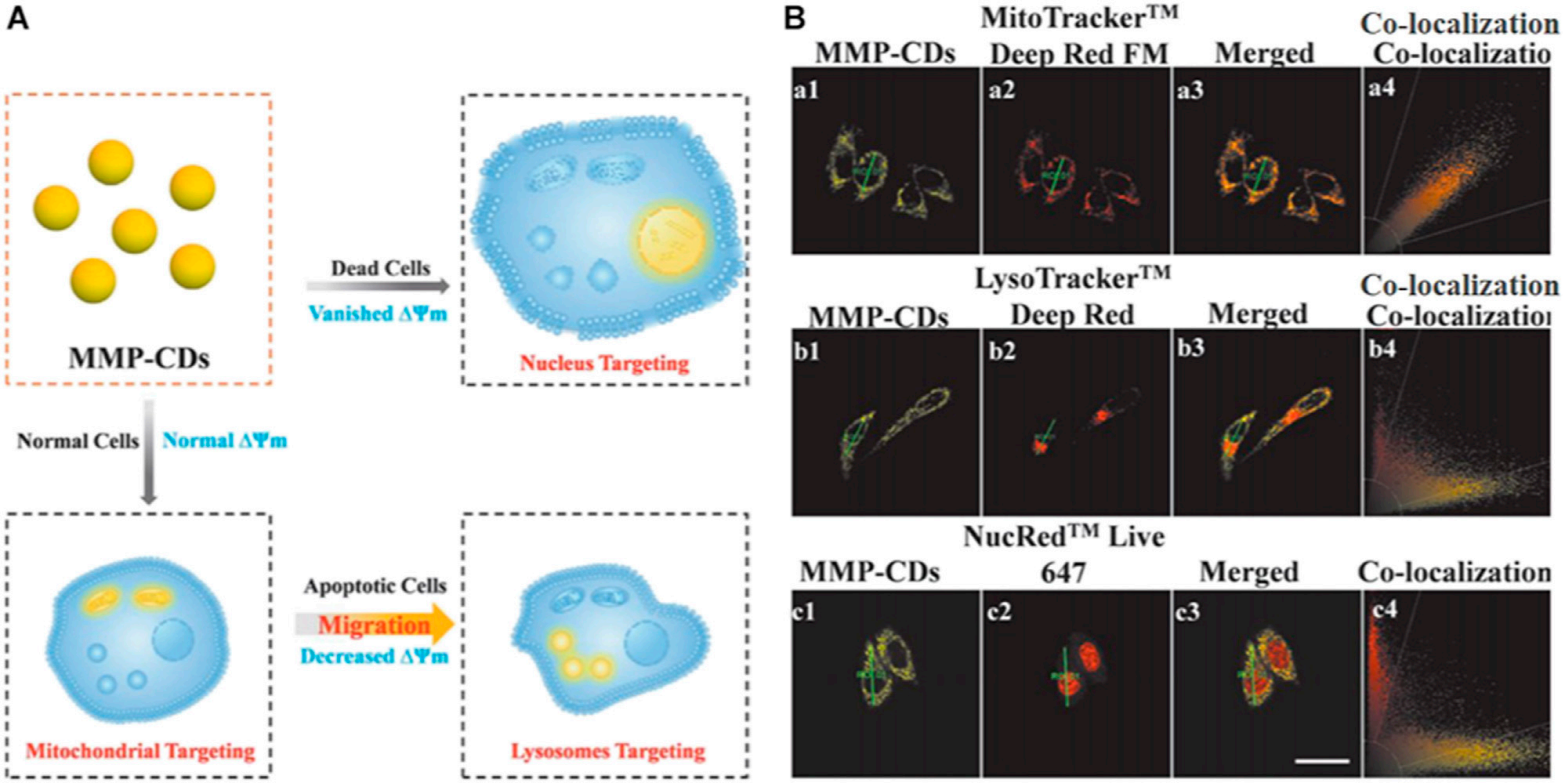
**(A)** Schematic diagram showing the sensing model of the CDs **(B)** Confocal images of HeLa cells costained with CDs and various trackers (MitoTracker Deep Red FM/LysoTracker Deep Red/NucRed Live 645), scale bar = 25 μm. Reproduced with permission from Yin et al., 2020. Copyright (2020), Wiley-VCH.

The enclosed environment or surface which acts as a partitioning provided by subcellular organelles could create space for the tailored chemical reactions of the organelles to perform specific functions. The subcellular migration of CDs enriches our understanding of how CDs interact with cells, shedding new light on the design of novel CDs for biomedical applications. Recently, Yin et al. synthesized CDs from 2,4-dihydroxybenzaldehyde and 1,2,3,3-tetramethyl-3H-indolium that were sensitive to the mitochondrial membrane potential (Figure 8) (Yin et al., 2020). The authors also found that the CDs displayed distinct targeting capabilities (mitochondria/lysosome/nucleus) at different mitochondrial membrane potential levels (normal/decreased/negligible) corresponding to three different degrees of cell viability (healthy/damaged/dead).

## Conclusion

This review presented a summary on recent advances in CD-based probes that can target specific organelles. These probes have been demonstrated to exhibit a variety of unique characteristics, such as high selectivity and sensitivity alongside low cytotoxicity, thus enabling their use as biological probes. In recent years, numerous probes have been designed with the ability to target key organelles, such as the nucleus, mitochondria, and lysosomes, thus providing valuable information about their activities. Such probes may offer several additional benefits as efficient drug delivery systems or nanocarriers. However, organelles such as the Golgi body and other secondary organelles have received little attention. By referring to the design of small-molecule fluorescent probes, a diverse range of organelle-targeting functional groups can be conjugated to CDs to enable their localization in specific organelles, and would help to develop a theragnostic nanomedicine for drug delivery and treatment applications for various diseases.

Although the field of CDs is still in its infancy, great advances can be anticipated in the future. For instance, CDs with NIR-I and even NIR-II emissions or up-conversion fluorescence property are still highly desired thus the need for further investigations. Moreover, there still exists some challenges with respect to subcellular imaging with CDs, such as realizing the visualization of numerous subcellular structures (microtubules, centrosome, actin filaments, and others) that still cannot be imaged with fluorescent CDs at present, the determination of the functions of proteins at the subcellular level, the correlations between fluctuations in the reactive species in organelles and the generation or progression of disease. Although low cytotoxicity of CDs has been reported from different research teams, standardized toxicity assessments methodologies are absent. Based on this situation, the comparison on the safety of CDs is still difficult. Therefore, there is the need for a systematic assessment of potential toxicity risks associated with the application of CDs so as to suitability for clinical applications such as in diagnosis and treatments. For better practical application in animal and clinical studies, CDs with high QYs and NIR-I and NIR-II emission are needed. It is expected that more high-performance CDs for subcellular targeted imaging and laboratory medicine on the subcellular scale will be developed in the near future. Bioimaging at the subcellular level would greatly increase our understanding of human biology and disease, and the development of CDs as a versatile tool for the prevention and treatment of disease shows enormous promise.

## References

[B1] Abeywickrama C. S. Wijesinghe K. J. Stahelin R. V. Pang Y. (2019). Red-emitting Pyrene-Benzothiazolium: Unexpected Selectivity to Lysosomes for Real-Time Cell Imaging without Alkalinizing Effect. Chem. Commun. 55, 3469–3472. 10.1039/C9CC01068H PMC6446231 30839045

[B2] Ahn J. Lee B. Choi Y. Jin H. Lim N. Y. Park J. (2018). Non-peptidic Guanidinium-Functionalized Silica Nanoparticles as Selective Mitochondria-Targeting Drug Nanocarriers. J. Mater. Chem. B 6, 5698–5707. 10.1039/C8TB01358F 32254976

[B3] Alamudi S. H. Satapathy R. Kim J. Su D. Ren H. Das R. (2016). Development of Background-free Tame Fluorescent Probes for Intracellular Live Cell Imaging. Nat. Commun. 7, 1–9. 10.1038/ncomms11964 PMC4915154 27321135

[B4] Baiceanu A. Mesdom P. Lagouge M. Foufelle F. (2016). Endoplasmic Reticulum Proteostasis in Hepatic Steatosis. Nat. Rev. Endocrinol. 12, 710–722. 10.1038/nrendo.2016.124 27516341

[B5] Bao Y.-W. Hua X.-W. Li Y.-H. Jia H.-R. Wu F.-G. (2019). Endoplasmic Reticulum-Targeted Phototherapy Using One-step Synthesized Trace Metal-Doped Carbon-Dominated Nanoparticles: Laser-Triggered Nucleolar Delivery and Increased Tumor Accumulation. Acta Biomater. 88, 462–476. 10.1016/j.actbio.2019.02.005 30735810

[B6] Bexiga M. Simpson J. (2013). Human Diseases Associated with Form and Function of the Golgi Complex. Ijms 14, 18670–18681. 10.3390/ijms140918670 24025425 PMC3794802

[B7] Biswas S. Dutta T. Silswal A. Bhowal R. Chopra D. Koner A. L. (2021). Strategic Engineering of Alkyl Spacer Length for a pH-Tolerant Lysosome Marker and Dual Organelle Localization. Chem. Sci. 12, 9630–9644. 10.1039/D1SC00542A 34349935 PMC8293980

[B8] Burke B. Stewart C. L. (2002). Life at the Edge: the Nuclear Envelope and Human Disease. Nat. Rev. Mol. Cel Biol 3, 575–585. 10.1038/nrm879 12154369

[B9] Butin-Israeli V. Adam S. A. Goldman A. E. Goldman R. D. (2012). Nuclear Lamin Functions and Disease. Trends Genet. 28, 464–471. 10.1016/j.tig.2012.06.001 22795640 PMC3633455

[B10] Cai Y. Shen H. Zhan J. Lin M. Dai L. Ren C. (2017). Supramolecular “Trojan Horse” for Nuclear Delivery of Dual Anticancer Drugs. J. Am. Chem. Soc. 139, 2876–2879. 10.1021/jacs.6b12322 28191948

[B11] Chan D. C. (2006). Mitochondria: Dynamic Organelles in Disease, Aging, and Development. Cell 125, 1241–1252. 10.1016/j.cell.2006.06.010 16814712

[B12] Chen S. Jia Y. Zou G.-Y. Yu Y.-L. Wang J.-H. (2019). A Ratiometric Fluorescent Nanoprobe Based on Naphthalimide Derivative-Functionalized Carbon Dots for Imaging Lysosomal Formaldehyde in HeLa Cells. Nanoscale 11, 6377–6383. 10.1039/C9NR00039A 30888365

[B13] Chen W. Yao Y. Chen T. Shen W. Tang S. Lee H. K. (2021). Application of Smartphone-Based Spectroscopy to Biosample Analysis: A Review. Biosens. Bioelectron. 172, 112788. 10.1016/j.bios.2020.112788 33157407

[B14] Chen X. Zhang X. Xia L. Y. Wang H. Y. Chen Z. Wu F. G. (2018). One-step Synthesis of Ultrasmall and Ultrabright Organosilica Nanodots with 100% Photoluminescence Quantum Yield: Long-Term Lysosome Imaging in Living, Fixed, and Permeabilized Cells. Nano Lett. 18, 1159–1167. 10.1021/acs.nanolett.7b04700 29368935

[B15] Cryan S.-A. McKiernan P. Cunningham C. M. Greene C. (2013). Targeting miRNA-Based Medicines to Cystic Fibrosis Airway Epithelial Cells Using Nanotechnology. Ijn 8, 3907. 10.2147/IJN.S47551 24143095 PMC3798151

[B16] D'Angelis do E. S. Barbosa C. Barbosa C. Corrêa J. R. Medeiros G. A. Barreto G. Magalhães K. G. (2015). Carbon Dots (C-Dots) from Cow Manure with Impressive Subcellular Selectivity Tuned by Simple Chemical Modification. Chem. Eur. J. 21, 5055–5060. 10.1002/chem.201406330 25693878

[B17] Dean A. C. R. Hinshelwood C. (1963). Integration of Cell Reactions. Nature 199, 7–11. 10.1038/199007a0 14047955

[B18] Derfus A. M. Chan W. C. W. Bhatia S. N. (2004). Intracellular Delivery of Quantum Dots for Live Cell Labeling and Organelle Tracking. Adv. Mater. 16, 961–966. 10.1002/adma.200306111

[B19] Dickinson B. C. Chang C. J. (2011). Chemistry and Biology of Reactive Oxygen Species in Signaling or Stress Responses. Nat. Chem. Biol. 7, 504–511. 10.1038/nchembio.607 21769097 PMC3390228

[B20] Ding H. Yu S.-B. Wei J.-S. Xiong H.-M. (2016). Full-color Light-Emitting Carbon Dots with a Surface-State-Controlled Luminescence Mechanism. ACS Nano 10, 484–491. 10.1021/acsnano.5b05406 26646584

[B21] Dong C. Liu Z. Liu J. Wu C. Neumann F. Wang H. (2016). A Highly Photostable Hyperbranched Polyglycerol-Based NIR Fluorescence Nanoplatform for Mitochondria-specific Cell Imaging. Adv. Healthc. Mater. 5, 2214–2226. 10.1002/adhm.201600212 27253762

[B22] Dong L. Krewson E. Yang L. (2017). Acidosis Activates Endoplasmic Reticulum Stress Pathways through GPR4 in Human Vascular Endothelial Cells. Ijms 18, 278. 10.3390/ijms18020278 PMC5343814 28134810

[B23] E S. Mao Q.-X. Yuan X.-L. Kong X.-L. Chen X.-W. Wang J.-H. (2018). Targeted Imaging of the Lysosome and Endoplasmic Reticulum and Their pH Monitoring with Surface Regulated Carbon Dots. Nanoscale 10, 12788–12796. 10.1039/C8NR03453B 29947397

[B24] Esteves da Silva J. C. G. Gonçalves H. M. R. (2011). Analytical and Bioanalytical Applications of Carbon Dots. Trac Trends Anal. Chem. 30, 1327–1336. 10.1016/j.trac.2011.04.009

[B25] Fan Z.-Y. Liu Z.-J. Zhang R.-L. Han G.-M. Zhang Z.-P. (2021). Preparation of Lysosome-Targeting Carbon Dots and its Application in Cell Imaging. Chin. J. Anal. Chem. 49, 1208–1217. 10.1016/S1872-2040(21)60108-1

[B26] Gao G. Jiang Y.-W. Yang J. Wu F.-G. (2017b). Mitochondria-targetable Carbon Quantum Dots for Differentiating Cancerous Cells from normal Cells. Nanoscale 9, 18368–18378. 10.1039/C7NR06764J 29143843

[B27] Gao M. X. Yang L. Zheng Y. Yang X. X. Zou H. Y. Han J. (2017a). “Click” on Alkynylated Carbon Quantum Dots: an Efficient Surface Functionalization for Specific Biosensing and Bioimaging. Chem. Eur. J. 23, 2171–2178. 10.1002/chem.201604963 27914103

[B28] Gao P. Pan W. Li N. Tang B. (2019). Fluorescent Probes for Organelle-Targeted Bioactive Species Imaging. Chem. Sci. 10, 6035–6071. 10.1039/C9SC01652J 31360411 PMC6585876

[B29] Gao P. Wang J. Zheng M. Xie Z. (2020). Lysosome Targeting Carbon Dots-Based Fluorescent Probe for Monitoring pH Changes *In Vitro* and *In Vivo* . Chem. Eng. J. 381, 122665. 10.1016/j.cej.2019.122665

[B30] Gauthier N. C. Rossier O. M. Mathur A. Hone J. C. Sheetz M. P. (2009). Plasma Membrane Area Increases with Spread Area by Exocytosis of a GPI-Anchored Protein Compartment. MBoC 20, 3261–3272. 10.1091/mbc.e09-01-0071 19458190 PMC2710839

[B31] Ge J. Lan M. Zhou B. Liu W. Guo L. Wang H. (2014). A Graphene Quantum Dot Photodynamic Therapy Agent with High Singlet Oxygen Generation. Nat. Commun. 5, 4596. 10.1038/ncomms5596 25105845 PMC4143951

[B32] Geng B. Yang D. Pan D. Wang L. Zheng F. Shen W. (2018). NIR-responsive Carbon Dots for Efficient Photothermal Cancer Therapy at Low Power Densities. Carbon 134, 153–162. 10.1016/j.carbon.2018.03.084

[B33] Geng X. Sun Y. Guo Y. Zhao Y. Zhang K. Xiao L. (2020). Fluorescent Carbon Dots for *In Situ* Monitoring of Lysosomal ATP Levels. Anal. Chem. 92, 7940–7946. 10.1021/acs.analchem.0c01335 32406677

[B34] Geng X. Sun Y. Li Z. Yang R. Zhao Y. Guo Y. (2019). Retrosynthesis of Tunable Fluorescent Carbon Dots for Precise Long‐Term Mitochondrial Tracking. Small 15, 1901517. 10.1002/smll.201901517 31165584

[B35] Gong N. Ma X. Ye X. Zhou Q. Chen X. Tan X. (2019). Carbon-dot-supported Atomically Dispersed Gold as a Mitochondrial Oxidative Stress Amplifier for Cancer Treatment. Nat. Nanotechnol. 14, 379–387. 10.1038/s41565-019-0373-6 30778211

[B36] Guo M. Xiang H.-J. Wang Y. Zhang Q.-L. An L. Yang S.-P. (2017). Ruthenium Nitrosyl Functionalized Graphene Quantum Dots as an Efficient Nanoplatform for NIR-Light-Controlled and Mitochondria-Targeted Delivery of Nitric Oxide Combined with Photothermal Therapy. Chem. Commun. 53, 3253–3256. 10.1039/c7cc00670e 28261712

[B37] Guo S. Sun Y. Geng X. Yang R. Xiao L. Qu L. (2020). Intrinsic Lysosomal Targeting Fluorescent Carbon Dots with Ultrastability for Long-Term Lysosome Imaging. J. Mater. Chem. B 8, 736–742. 10.1039/C9TB02043H 31894833

[B38] Han G. Zhao J. Zhang R. Tian X. Liu Z. Wang A. (2019). Membrane‐Penetrating Carbon Quantum Dots for Imaging Nucleic Acid Structures in Live Organisms. Angew. Chem. Int. Ed. 58, 7087–7091. 10.1002/anie.201903005 30912239

[B39] He H. Wang Z. Cheng T. Liu X. Wang X. Wang J. (2016). Visible and Near-Infrared Dual-Emission Carbogenic Small Molecular Complex with High RNA Selectivity and Renal Clearance for Nucleolus and Tumor Imaging. ACS Appl. Mater. Inter. 8, 28529–28537. 10.1021/acsami.6b10737 27704754

[B40] He Y. Li Z. Jia Q. Shi B. Zhang H. Wei L. (2017). Ratiometric Fluorescent Detection of Acidic pH in Lysosome with Carbon Nanodots. Chin. Chem. Lett. 28, 1969–1974. 10.1016/j.cclet.2017.07.027

[B41] Hua X.-W. Bao Y.-W. Chen Z. Wu F.-G. (2017). Carbon Quantum Dots with Intrinsic Mitochondrial Targeting Ability for Mitochondria-Based Theranostics. Nanoscale 9, 10948–10960. 10.1039/C7NR03658B 28736787

[B42] Hua X.-W. Bao Y.-W. Wu F.-G. (2018b). Fluorescent Carbon Quantum Dots with Intrinsic Nucleolus-Targeting Capability for Nucleolus Imaging and Enhanced Cytosolic and Nuclear Drug Delivery. ACS Appl. Mater. Inter. 10, 10664–10677. 10.1021/acsami.7b19549 29508612

[B43] Hua X.-W. Bao Y.-W. Zeng J. Wu F.-G. (2019). Nucleolus-targeted Red Emissive Carbon Dots with Polarity-Sensitive and Excitation-independent Fluorescence Emission: High-Resolution Cell Imaging and *In Vivo* Tracking. ACS Appl. Mater. Inter. 11, 32647–32658. 10.1021/acsami.9b09590 31381288

[B44] Hua X.-W. Bao Y.-W. Zeng J. Wu F.-G. (2018a). Ultrasmall All-In-One Nanodots Formed via Carbon Dot-Mediated and Albumin-Based Synthesis: Multimodal Imaging-Guided and Mild Laser-Enhanced Cancer Therapy. ACS Appl. Mater. Inter. 10, 42077–42087. 10.1021/acsami.8b16065 30403472

[B45] James A. M. Sharpley M. S. Manas A.-R. B. Frerman F. E. Hirst J. Smith R. A. J. (2007). Interaction of the Mitochondria-Targeted Antioxidant mitoQ with Phospholipid Bilayers and Ubiquinone Oxidoreductases. J. Biol. Chem. 282, 14708–14718. 10.1074/jbc.M611463200 17369262

[B46] Jia X. Pei M. Zhao X. Tian K. Zhou T. Liu P. (2016). PEGylated Oxidized Alginate-DOX Prodrug Conjugate Nanoparticles Cross-Linked with Fluorescent Carbon Dots for Tumor Theranostics. ACS Biomater. Sci. Eng. 2, 1641–1648. 10.1021/acsbiomaterials.6b00443 33440597

[B47] Jung Y. K. Shin E. Kim B.-S. (2015). Cell Nucleus-Targeting Zwitterionic Carbon Dots. Sci. Rep. 5, 18807. 10.1038/srep18807 26689549 PMC4686939

[B48] Li B. Gong D. Li X. Zhang L. Dong Y. Li W. (2018). Subcellular Fluorescence Imaging for BHK Cell and Multiple Sensing Based on Carbon Dots with Two strong Emission Peaks. Sensors Actuators B: Chem. 258, 757–765. 10.1016/j.snb.2017.11.175

[B49] Li C. H. Li R. S. Li C. M. Huang C. Z. Zhen S. J. (2019). Precise Ricin A-Chain Delivery by Golgi-Targeting Carbon Dots. Chem. Commun. 55, 6437–6440. 10.1039/C9CC01599J 31095140

[B50] Li D. Lin L. Fan Y. Liu L. Shen M. Wu R. (2021). Ultrasound-enhanced Fluorescence Imaging and Chemotherapy of Multidrug-Resistant Tumors Using Multifunctional Dendrimer/carbon Dot Nanohybrids. Bioactive Mater. 6, 729–739. 10.1016/j.bioactmat.2020.09.015 PMC7519212 33024894

[B51] Li R. S. Gao P. F. Zhang H. Z. Zheng L. L. Li C. M. Wang J. (2017). Chiral Nanoprobes for Targeting and Long-Term Imaging of the Golgi Apparatus. Chem. Sci. 8, 6829–6835. 10.1039/C7SC01316G 29147508 PMC5643954

[B52] Lin F. Bao Y.-W. Wu F.-G. (2018). Improving the Phototherapeutic Efficiencies of Molecular and Nanoscale Materials by Targeting Mitochondria. Molecules 23, 3016. 10.3390/molecules23113016 PMC6278291 30453692

[B53] Lin M. T. Beal M. F. (2006). Mitochondrial Dysfunction and Oxidative Stress in Neurodegenerative Diseases. Nature 443, 787–795. 10.1038/nature05292 17051205

[B54] Liu C. Gao X. Yuan J. Zhang R. (202011609). Advances in the Development of Fluorescence Probes for Cell Plasma Membrane Imaging. Trac Trends Anal. Chem. 133, 116092. 10.1016/j.trac.2020.116092

[B55] Liu H. Sun Y. Li Z. Yang J. Aryee A. A. Qu L. (2019b). Lysosome-targeted Carbon Dots for Ratiometric Imaging of Formaldehyde in Living Cells. Nanoscale 11, 8458–8463. 10.1039/C9NR01678C 30994690

[B56] Liu H. Yang J. Li Z. Xiao L. Aryee A. A. Sun Y. (2019a). Hydrogen-Bond-Induced Emission of Carbon Dots for Wash-free Nucleus Imaging. Anal. Chem. 91, 9259–9265. 10.1021/acs.analchem.9b02147 31204808

[B57] Lodish H. Berk A. Kaiser C. A. Krieger M. Scott M. P. Bretscher A. (2008). Molecular Cell Biology. Macmillan.

[B58] Lovrić J. Cho S. J. Winnik F. M. Maysinger D. (2005). Unmodified Cadmium telluride Quantum Dots Induce Reactive Oxygen Species Formation Leading to Multiple Organelle Damage and Cell Death. Chem. Biol. 12, 1227–1234. 10.1016/j.chembiol.2005.09.008 16298302

[B59] Mao Q.-X. E S. Xia J.-M. Song R.-S. Shu Y. Chen X.-W. (2016). Hydrophobic Carbon Nanodots with Rapid Cell Penetrability and Tunable Photoluminescence Behavior for *In Vitro* and *In Vivo* Imaging. Langmuir 32, 12221–12229. 10.1021/acs.langmuir.6b03331 27805819

[B60] Matai I. Sachdev A. Gopinath P. (2015). Self-assembled Hybrids of Fluorescent Carbon Dots and PAMAM Dendrimers for Epirubicin Delivery and Intracellular Imaging. ACS Appl. Mater. Inter. 7, 11423–11435. 10.1021/acsami.5b02095 25946165

[B61] Mindell J. A. (2012). Lysosomal Acidification Mechanisms. Annu. Rev. Physiol. 74, 69–86. 10.1146/annurev-physiol-012110-142317 22335796

[B62] Modica-Napolitano J. S. Aprille J. R. (2001). Delocalized Lipophilic Cations Selectively Target the Mitochondria of Carcinoma Cells. Adv. Drug Deliv. Rev. 49, 63–70. 10.1016/s0169-409x(01)00125-9 11377803

[B63] Modica-Napolitano J. S. Singh K. K. (2004). Mitochondrial Dysfunction in Cancer. Mitochondrion 4, 755–762. 10.1016/j.mito.2004.07.027 16120430

[B64] Mohammadinejad R. Dadashzadeh A. Moghassemi S. Ashrafizadeh M. Dehshahri A. Pardakhty A. (2019). Shedding Light on Gene Therapy: Carbon Dots for the Minimally Invasive Image-Guided Delivery of Plasmids and Noncoding RNAs - A Review. J. Adv. Res. 18, 81–93. 10.1016/j.jare.2019.01.004 30828478 PMC6383136

[B65] Mukherjee A. Saha P. C. Das R. S. Bera T. Guha S. (2021). Acidic pH-Activatable Visible to Near-Infrared Switchable Ratiometric Fluorescent Probe for Live-Cell Lysosome Targeted Imaging. ACS Sens. 6, 2141–2146. 10.1021/acssensors.1c00961 34125510

[B66] Newmeyer D. D. Ferguson-Miller S. (2003). Mitochondria. Cell 112, 481–490. 10.1016/s0092-8674(03)00116-8 12600312

[B67] Papanicolaou G. N. Traut H. F. (1997). The Diagnostic Value of Vaginal Smears in Carcinoma of the Uterus. 1941. Arch. Pathol. Lab. Med. 121, 211–224. 9111103

[B68] Pei M. Jia X. Liu P. (2018). Design of Janus-like PMMA-PEG-FA Grafted Fluorescent Carbon Dots and Their Nanoassemblies for Leakage-free Tumor Theranostic Application. Mater. Des. 155, 288–296. 10.1016/j.matdes.2018.06.007

[B69] Qin H. Sun Y. Geng X. Zhao K. Meng H. Yang R. (2020). A Wash-free Lysosome Targeting Carbon Dots for Ultrafast Imaging and Monitoring Cell Apoptosis Status. Analytica Chim. Acta 1106, 207–215. 10.1016/j.aca.2020.02.002 32145850

[B70] Reddy K. L. Feinberg A. P. (2013). Higher Order Chromatin Organization in Cancer. Semin. Cancer Biol. 23, 109–115. 10.1016/j.semcancer.2012.12.001 23266653 PMC3715089

[B71] Ronzani C. Van Belle C. Didier P. Spiegelhalter C. Pierrat P. Lebeau L. (2019). Lysosome Mediates Toxicological Effects of Polyethyleneimine-Based Cationic Carbon Dots. J. Nanopart Res. 21, 4. 10.1007/s11051-018-4438-5

[B72] Ross M. F. Filipovska A. Smith R. A. J. Gait M. J. Murphy M. P. (2004). Cell-penetrating Peptides Do Not Cross Mitochondrial Membranes Even when Conjugated to a Lipophilic Cation: Evidence against Direct Passage through Phospholipid Bilayers. Biochem. J. 383, 457–468. 10.1042/BJ20041095 15270716 PMC1133738

[B73] Roy E. Patra S. Madhuri R. Sharma P. K. (2017). RETRACTED: Carbon Dot/TAT Peptide Co-conjugated Bubble Nanoliposome for Multicolor Cell Imaging, Nuclear-Targeted Delivery, and Chemo/photothermal Synergistic Therapy. Chem. Eng. J. 312, 144–157. 10.1016/j.cej.2016.11.122

[B74] Singh H. Sreedharan S. Tiwari K. Green N. H. Smythe C. Pramanik S. K. (2019). Two Photon Excitable Graphene Quantum Dots for Structured Illumination Microscopy and Imaging Applications: Lysosome Specificity and Tissue-dependent Imaging. Chem. Commun. 55, 521–524. 10.1039/C8CC08610A 30556083

[B75] Stephens A. D. Liu P. Z. Banigan E. J. Almassalha L. M. Backman V. Adam S. A. (2018). Chromatin Histone Modifications and Rigidity Affect Nuclear Morphology Independent of Lamins. MBoC 29, 220–233. 10.1091/mbc.E17-06-0410 29142071 PMC5909933

[B76] Strzyz P. (2021). Lysosome Transport Interrupted. Nat. Rev. Mol. Cel Biol 22, 373. 10.1038/s41580-021-00376-4 33911233

[B77] Su J. H. Anderson A. J. Cummings B. J. Cotman C. W. (1994). Immunohistochemical Evidence for Apoptosis in Alzheimerʼs Disease. Neuroreport 5, 2529–2533. 10.1097/00001756-199412000-00031 7696596

[B78] Sun Y. Qin H. Geng X. Yang R. Qu L. Kani A. N. (2020). Rational Design of Far-Red to Near-Infrared Emitting Carbon Dots for Ultrafast Lysosomal Polarity Imaging. ACS Appl. Mater. Inter. 12, 31738–31744. 10.1021/acsami.0c05005 32608958

[B79] Tong L. Wang X. Chen Z. Liang Y. Yang Y. Gao W. (2020). One-Step Fabrication of Functional Carbon Dots with 90% Fluorescence Quantum Yield for Long-Term Lysosome Imaging. Anal. Chem. 92, 6430–6436. 10.1021/acs.analchem.9b05553 32268724

[B80] Vankayala R. Kuo C.-L. Nuthalapati K. Chiang C.-S. Hwang K. C. (2015). Nucleus-targeting Gold Nanoclusters for Simultaneous *In Vivo* Fluorescence Imaging, Gene Delivery, and NIR-Light Activated Photodynamic Therapy. Adv. Funct. Mater. 25, 5934–5945. 10.1002/adfm.201502650

[B81] Villanueva D. G. Mendiola M. R. Nguyen H. X. Weissig V. (2015). Influence of Triphenylphosphonium (TPP) Cation Hydrophobization with Phospholipids on Cellular Toxicity and Mitochondrial Selectivity. Sojpps 2, 1–9. 10.15226/2374-6866/2/1/00121

[B82] Walker J. E. Dickson V. K. (2006). The Peripheral Stalk of the Mitochondrial ATP Synthase. Biochim. Biophys. Acta (Bba) - Bioenerg. 1757, 286–296. 10.1016/j.bbabio.2006.01.001 16697972

[B83] Wan Q. Chen S. Shi W. Li L. Ma H. (2014). Lysosomal pH Rise during Heat Shock Monitored by a Lysosome-Targeting Near-Infrared Ratiometric Fluorescent Probe. Angew. Chem. Int. Ed. 53, 10916–10920. 10.1002/anie.201405742 25154475

[B84] Wang B. Wang Y. Wu H. Song X. Guo X. Zhang D. (2014). A Mitochondria-Targeted Fluorescent Probe Based on TPP-Conjugated Carbon Dots for Both One- and Two-Photon Fluorescence Cell Imaging. RSC Adv. 4, 49960–49963. 10.1039/C4RA07467J

[B85] Wang C. Wu C. Zhou X. Han T. Xin X. Wu J. (2013). Enhancing Cell Nucleus Accumulation and DNA Cleavage Activity of Anti-cancer Drug via Graphene Quantum Dots. Sci. Rep. 3, 2852. 10.1038/srep02852 24092333 PMC3790198

[B86] Wang H.-J. He X. Luo T.-Y. Zhang J. Liu Y.-H. Yu X.-Q. (2017). Amphiphilic Carbon Dots as Versatile Vectors for Nucleic Acid and Drug Delivery. Nanoscale 9, 5935–5947. 10.1039/C7NR01029J 28440819

[B87] Wang L. Wu B. Li W. Wang S. Li Z. Li M. (2018). Amphiphilic Graphene Quantum Dots as Self-Targeted Fluorescence Probes for Cell Nucleus Imaging. Adv. Biosys. 2, 1700191. 10.1002/adbi.201700191

[B88] Wang X. Qu K. Xu B. Ren J. Qu X. (2011). Multicolor Luminescent Carbon Nanoparticles: Synthesis, Supramolecular Assembly with Porphyrin, Intrinsic Peroxidase-like Catalytic Activity and Applications. Nano Res. 4, 908–920. 10.1007/s12274-011-0147-4

[B89] Weissmann G. (1967). The Role of Lysosomes in Inflammation and Disease. Annu. Rev. Med. 18, 97–112. 10.1146/annurev.me.18.020167.000525 5337539

[B90] Wu H. Pang L.-F. Wei N. Guo X.-F. Wang H. (2021). Nucleus-targeted N-Doped Carbon Dots via DNA-Binding for Imaging of Hypochlorous in Cells and Zebrafish. Sensors Actuators B: Chem. 333, 129626. 10.1016/j.snb.2021.129626

[B91] Wu L. Li X. Ling Y. Huang C. Jia N. (2017b). Morpholine Derivative-Functionalized Carbon Dots-Based Fluorescent Probe for Highly Selective Lysosomal Imaging in Living Cells. ACS Appl. Mater. Inter. 9, 28222–28232. 10.1021/acsami.7b08148 28787116

[B92] Wu X. Sun S. Wang Y. Zhu J. Jiang K. Leng Y. (2017a). A Fluorescent Carbon-Dots-Based Mitochondria-Targetable Nanoprobe for Peroxynitrite Sensing in Living Cells. Biosens. Bioelectron. 90, 501–507. 10.1016/j.bios.2016.10.060 27825883

[B93] Xiang H.-J. Deng Q. An L. Guo M. Yang S.-P. Liu J.-G. (2016a). Tumor Cell Specific and Lysosome-Targeted Delivery of Nitric Oxide for Enhanced Photodynamic Therapy Triggered by 808 Nm Near-Infrared Light. Chem. Commun. 52, 148–151. 10.1039/C5CC07006F 26503188

[B94] Xiang H.-J. Guo M. An L. Yang S.-P. Zhang Q.-L. Liu J.-G. (2016b). A Multifunctional Nanoplatform for Lysosome Targeted Delivery of Nitric Oxide and Photothermal Therapy under 808 Nm Near-Infrared Light. J. Mater. Chem. B 4, 4667–4674. 10.1039/C6TB00730A 32263238

[B95] Xiang J.-F. Liu Y.-X. Sun D. Zhang S.-J. Fu Y.-L. Zhang X.-H. (2012). Synthesis, Spectral Properties of Rhodanine Complex Merocyanine Dyes as Well as Their Effect on K562 Leukemia Cells. Dyes Pigm. 93, 1481–1487. 10.1016/j.dyepig.2011.10.017

[B96] Xu P. Van Kirk E. A. Zhan Y. Murdoch W. J. Radosz M. Shen Y. (2007). Targeted Charge-Reversal Nanoparticles for Nuclear Drug Delivery. Angew. Chem. Int. Ed. 46, 4999–5002. 10.1002/anie.200605254 17526044

[B97] Xu W. Zeng Z. Jiang J.-H. Chang Y.-T. Yuan L. (2016). Discerning the Chemistry in Individual Organelles with Small-Molecule Fluorescent Probes. Angew. Chem. Int. Ed. 55, 13658–13699. 10.1002/anie.201510721 27571316

[B98] Yang L. Wang Z. Wang J. Jiang W. Jiang X. Bai Z. (2016). Doxorubicin Conjugated Functionalizable Carbon Dots for Nucleus Targeted Delivery and Enhanced Therapeutic Efficacy. Nanoscale 8, 6801–6809. 10.1039/C6NR00247A 26957191

[B99] Yang X.-D. Xiang H.-J. An L. Yang S.-P. Liu J.-G. (2015). Targeted Delivery of Photoactive Diazido PtIV Complexes Conjugated with Fluorescent Carbon Dots. New J. Chem. 39, 800–804. 10.1039/C4NJ01758G

[B100] Yang Y. Hu Y. Shi W. Ma H. (2020). A Near-Infrared Fluorescence Probe for Imaging of Pantetheinase in Cells and Mice *In Vivo* . Chem. Sci. 11, 12802–12806. 10.1039/D0SC04537C 34123238 PMC8163316

[B101] Yang Y. Wang S. Chen S. Shen Y. Zhu M. (2018). Switching the Subcellular Organelle Targeting of Atomically Precise Gold Nanoclusters by Modifying the Capping Ligand. Chem. Commun. 54, 9222–9225. 10.1039/C8CC04474K 30066001

[B102] Yao C. Tu Y. Ding L. Li C. Wang J. Fang H. (2017). Tumor Cell-specific Nuclear Targeting of Functionalized Graphene Quantum Dots *In Vivo* . Bioconjug. Chem. 28, 2608–2619. 10.1021/acs.bioconjchem.7b00466 28903003

[B103] Yao H. Su L. Zeng M. Cao L. Zhao W. Chen C. (2016). Construction of Magnetic-Carbon-Quantum-Dots-Probe-Labeled Apoferritin Nanocages for Bioimaging and Targeted Therapy. Ijn Vol. 11, 4423–4438. 10.2147/IJN.S108039 PMC5019280 27660437

[B104] Yin X. Sun Y. Geng X. Li J. Li J. Yang R. (2020). Spatiotemporally Monitoring Cell Viability through Programmable Mitochondrial Membrane Potential Transformation by Using Fluorescent Carbon Dots. Adv. Biosys. 4, 1900261. 10.1002/adbi.201900261 32293145

[B105] Yu F. Chen Z. Wang B. Jin Z. Hou Y. Ma S. (2016). The Role of Lysosome in Cell Death Regulation. Tumor Biol. 37, 1427–1436. 10.1007/s13277-015-4516-6 26631036

[B106] Yuan F. Li S. Fan Z. Meng X. Fan L. Yang S. H. (2016). Shining Carbon Dots: Synthesis and Biomedical and Optoelectronic Applications. Nano Today 11, 565–586. 10.1016/j.nantod.2016.08.006

[B107] Yuan M. Guo Y. Wei J. Li J. Long T. Liu Z. (2017b). Optically Active Blue-Emitting Carbon Dots to Specifically Target the Golgi Apparatus. RSC Adv. 7, 49931–49936. 10.1039/C7RA09271G

[B108] Yuan Y. Guo B. Hao L. Liu N. Lin Y. Guo W. (2017a). Doxorubicin-loaded Environmentally Friendly Carbon Dots as a Novel Drug Delivery System for Nucleus Targeted Cancer Therapy. Colloids Surf. B: Biointerfaces 159, 349–359. 10.1016/j.colsurfb.2017.07.030 28806666

[B109] Zhang K. Kaufman R. J. (2008). From Endoplasmic-Reticulum Stress to the Inflammatory Response. Nature 454, 455–462. 10.1038/nature07203 18650916 PMC2727659

[B110] Zhang Q. Q. Yang T. Li R. S. Zou H. Y. Li Y. F. Guo J. (2018). A Functional Preservation Strategy for the Production of Highly Photoluminescent Emerald Carbon Dots for Lysosome Targeting and Lysosomal pH Imaging. Nanoscale 10, 14705–14711. 10.1039/C8NR03212B 30039824

[B111] Zhang Y. Shen Y. Teng X. Yan M. Bi H. Morais P. C. (2015). Mitochondria-targeting Nanoplatform with Fluorescent Carbon Dots for Long Time Imaging and Magnetic Field-Enhanced Cellular Uptake. ACS Appl. Mater. Inter. 7, 10201–10212. 10.1021/acsami.5b00405 25942702

[B112] Zhang Y. Zhang C. Chen J. Liu L. Hu M. Li J. (2017). Trackable Mitochondria-Targeting Nanomicellar Loaded with Doxorubicin for Overcoming Drug Resistance. ACS Appl. Mater. Inter. 9, 25152–25163. 10.1021/acsami.7b07219 28697306

[B113] Zheng M. Li Y. Li Y. Liu S. Wang W. Xie Z. (2016). One-pot to Synthesize Multifunctional Carbon Dots for Near Infrared Fluorescence Imaging and Photothermal Cancer Therapy. ACS Appl. Mater. Inter. 8, 23533–23541. 10.1021/acsami.6b07453 27558196

[B114] Zhou D. Huang H. Wang Y. Wang Y. Hu Z. Li X. (2019). A Yellow-Emissive Carbon Nanodot-Based Ratiometric Fluorescent Nanosensor for Visualization of Exogenous and Endogenous Hydroxyl Radicals in the Mitochondria of Live Cells. J. Mater. Chem. B 7, 3737–3744. 10.1039/C9TB00289H

[B115] Zhou N. Zhu S. Maharjan S. Hao Z. Song Y. Zhao X. (2014). Elucidating the Endocytosis, Intracellular Trafficking, and Exocytosis of Carbon Dots in Neural Cells. RSC Adv. 4, 62086–62095. 10.1039/C4RA09525A

[B116] Zhu H. Fan J. Du J. Peng X. (2016). Fluorescent Probes for Sensing and Imaging within Specific Cellular Organelles. Acc. Chem. Res. 49, 2115–2126. 10.1021/acs.accounts.6b00292 27661761

[B117] Zhu Z. Li Q. Li P. Xun X. Zheng L. Ning D. (2019). Surface Charge Controlled Nucleoli Selective Staining with Nanoscale Carbon Dots. PLoS ONE 14, e0216230. 10.1371/journal.pone.0216230 31150413 PMC6544201

[B118] Zlitni A. Gowrishankar G. Steinberg I. Haywood T. Sam Gambhir S. (2020). Maltotriose-based Probes for Fluorescence and Photoacoustic Imaging of Bacterial Infections. Nat. Commun. 11, 1–13. 10.1038/s41467-020-14985-8 32144257 PMC7060353

